# Label-free, multi-parametric assessments of cell metabolism and matrix remodeling within human and early-stage murine osteoarthritic articular cartilage

**DOI:** 10.1038/s42003-023-04738-w

**Published:** 2023-04-13

**Authors:** Zhiyi Liu, Carrie K. Hui Mingalone, Einstein Gnanatheepam, Judith M. Hollander, Yang Zhang, Jia Meng, Li Zeng, Irene Georgakoudi

**Affiliations:** 1 Department of Biomedical Engineering, Tufts University, Medford, MA 02155 USA; 2 Program in Cell, Molecular, and Developmental Biology, Graduate School of Biomedical Sciences, Tufts University, Boston, MA 02111 USA; 3 State Key Laboratory of Modern Optical Instrumentation, College of Optical Science and Engineering, Zhejiang University, Hangzhou, Zhejiang 310027 China; 4 Department of Immunology, Tufts University School of Medicine, Boston, MA 02111 USA; 5 Department of Orthopaedics, Tufts Medical Center, Boston, MA 02111 USA; 6 Present Address: State Key Laboratory of Modern Optical Instrumentation, College of Optical Science and Engineering; International Research Center for Advanced Photonics, Zhejiang University, Hangzhou, Zhejiang 310027 China; 7 Present Address: Intelligent Optics & Photonics Research Center, Jiaxing Research Institute, Zhejiang University, Jiaxing, Zhejiang 314000 China

**Keywords:** Osteoarthritis, Optical imaging

## Abstract

Osteoarthritis (OA) is characterized by the progressive deterioration of articular cartilage, involving complicated cell-matrix interactions. Systematic investigations of dynamic cellular and matrix changes during OA progression are lacking. In this study, we use label-free two-photon excited fluorescence (TPEF) and second harmonic generation (SHG) imaging to assess cellular and extracellular matrix features of murine articular cartilage during several time points at early stages of OA development following destabilization of medial meniscus surgery. We detect significant changes in the organization of collagen fibers and crosslink-associated fluorescence of the superficial zone as early as one week following surgery. Such changes become significant within the deeper transitional and radial zones at later time-points, highlighting the importance of high spatial resolution. Cellular metabolic changes exhibit a highly dynamic behavior, and indicate metabolic reprogramming from enhanced oxidative phosphorylation to enhanced glycolysis or fatty acid oxidation over the ten-week observation period. The optical metabolic and matrix changes detected within this mouse model are consistent with differences identified in excised human cartilage specimens from OA and healthy cartilage specimens. Thus, our studies reveal important cell-matrix interactions at the onset of OA that may enable improved understanding of OA development and identification of new potential treatment targets.

## Abstract (web summary)

Label-free two-photon excited fluorescence (TPEF) and second harmonic generation (SHG) imaging of osteoarthritic articular cartilage from patients and mice reveal the importance of cell-matrix interactions at disease onset.

## Introduction

Osteoarthritis (OA), a chronic degenerative joint disorder characterized by articular cartilage destruction and osteophyte formation^1^, is the most common form of arthritis and affects millions of people worldwide^2^. However, the mechanism underlying OA pathogenesis remains largely elusive, and current treatments for OA generally rely on symptom relief, instead of disease modification^3^. In order to design strategies to interfere with OA progression, it is critical to understand the pathways that lead to the initiation and development of this disease, whose etiology appears to bridge biomechanics and biochemistry^3^. Among the numerous alterations induced by OA, changes in cellular metabolism, and spatial organization and cross-linking of extracellular matrix (ECM) are very important aspects; but they are typically detected at end-stage OA^4–6^, as they are likely subtle and heterogeneous during early stages, and, thus, difficult to characterize using established tools. Therefore, sensitive approaches for the detection of these changes are needed.

In the past few years, several studies have demonstrated that cellular metabolic function is drastically altered in OA and aberrant immunometabolism may be a key feature of many phenotypes of OA^7^. High-resolution fluorescence imaging-based approaches, that rely on exogenous fluorescent probes targeting specific cellular organelles or proteins during OA progression^8^, require cellular manipulations and can be confounded by artifacts related to the distribution of the fluorophores. Two-photon excited fluorescence (TPEF) has emerged as a powerful modality for high-resolution, label-free quantitative assessments of metabolic activity^9,10^. TPEF imaging relies on the simultaneous absorption of two low energy, infra-red photons, exciting the fluorophore and leading to the emission of a single higher energy photon in the visible range of the spectrum. Light is delivered in the form of focused, highly intense, but very short (~100 fs) pulses, to overcome the low-probability of two-photon excitation, while maintaining average illumination powers at safe levels. Micron level depth resolution in three-dimensional (3D) specimens that extend several hundred microns is a key advantage of this imaging approach. Two key coenzymes actively involved in several important metabolic pathways, NAD(P)H and FAD, provide endogenous fluorescence, enabling label-free metabolic assessments^11,12^. Several optical biomarkers have been developed that rely on analysis of their intensity and lifetime characteristics for metabolic assessments. These include the optical redox ratio, defined in our studies as the TPEF intensity of FAD/[NAD(P)H + FAD]^13,14^, the NAD(P)H long lifetime intensity fraction (LLIF) extracted from fluorescence lifetime analysis^15,16^, and the level of mitochondrial clustering or fragmentation, which reflects the balance of mitochondrial fusion and fission levels for optimizing energy production and delivery^17–19^. We have demonstrated that the optical redox ratio linearly correlates with the biochemical redox ratio NAD^+^/(NAD^+^+NADH) that is typically measured using liquid chromatography-mass spectrometry^13,14^. Moreover, the calculated mitochondrial clustering levels in response to typical metabolic perturbations have been validated to be consistent with observations from high-resolution imaging of mitochondria using stains and perturbations such as hypoxia, established to induce mitochondrial fragmentation in several studies^14,18^.

In addition to metabolic changes, there are subtle changes in the cartilage matrix in early OA, highlighted by modifications in the spatial organization of collagen fibers^20^. In normal articular cartilage, 90% of the collagen content is type II collagen (Col II)^21^. Second harmonic generation (SHG) microscopy enables effective label-free imaging of collagen fibers in 3D biological tissues at sub-micron resolution^21–23^. SHG relies on non-linear scattering, which yields the generation of a single high energy scattered photon at half the wavelength of the two incident photons that simultaneously interact with the scatterer. SHG signals are generated only from scatterers that have a non-centrosymmetric structure, such as collagen fibers. Besides alterations in matrix organization, previous studies have also demonstrated that changes in the biophysical properties of the chondrocyte microenvironment triggered by cartilage matrix cross-linking play a causal role in OA pathogenesis^24^. We have shown that the endogenous collagen-associated TPEF signal at specific excitation-emission settings is highly correlated with mass spectrometry-based assessments of lysyl-oxidase-mediated collagen cross-links^25^. Besides fluorescence intensity, it has been reported that the fluorescence lifetime of cross-links is altered with cartilage matrix degradation^6^, revealing that the fluorescence lifetime of cross-links could potentially serve as an indicator for OA as well.

In this study, we sought to exploit the wealth of complementary information that can be extracted from combined label-free TPEF and SHG images to assess simultaneously changes that occur within the cellular and ECM components at the onset of OA and gain improved understanding of the dynamics of potential interactions. We imaged mouse knee cryosections acquired at several time points within the first ten weeks following OA-inducing surgery (destabilization of medial meniscus, DMM) and corresponding sham surgery of the other knee. We quantified the 3D collagen fiber organization and associated cross-linking fluorescence intensity and lifetime along with chondrocyte metabolic function and characterized corresponding changes over time. Finally, we compared our findings to similar assessments performed using excised human cartilage specimens from OA-affected and unaffected regions to highlight the translational relevance of our results.

## Results

### Multi-modal, label-free two-photon imaging and segmentation of mouse articular cartilage

**Fig. 1:**
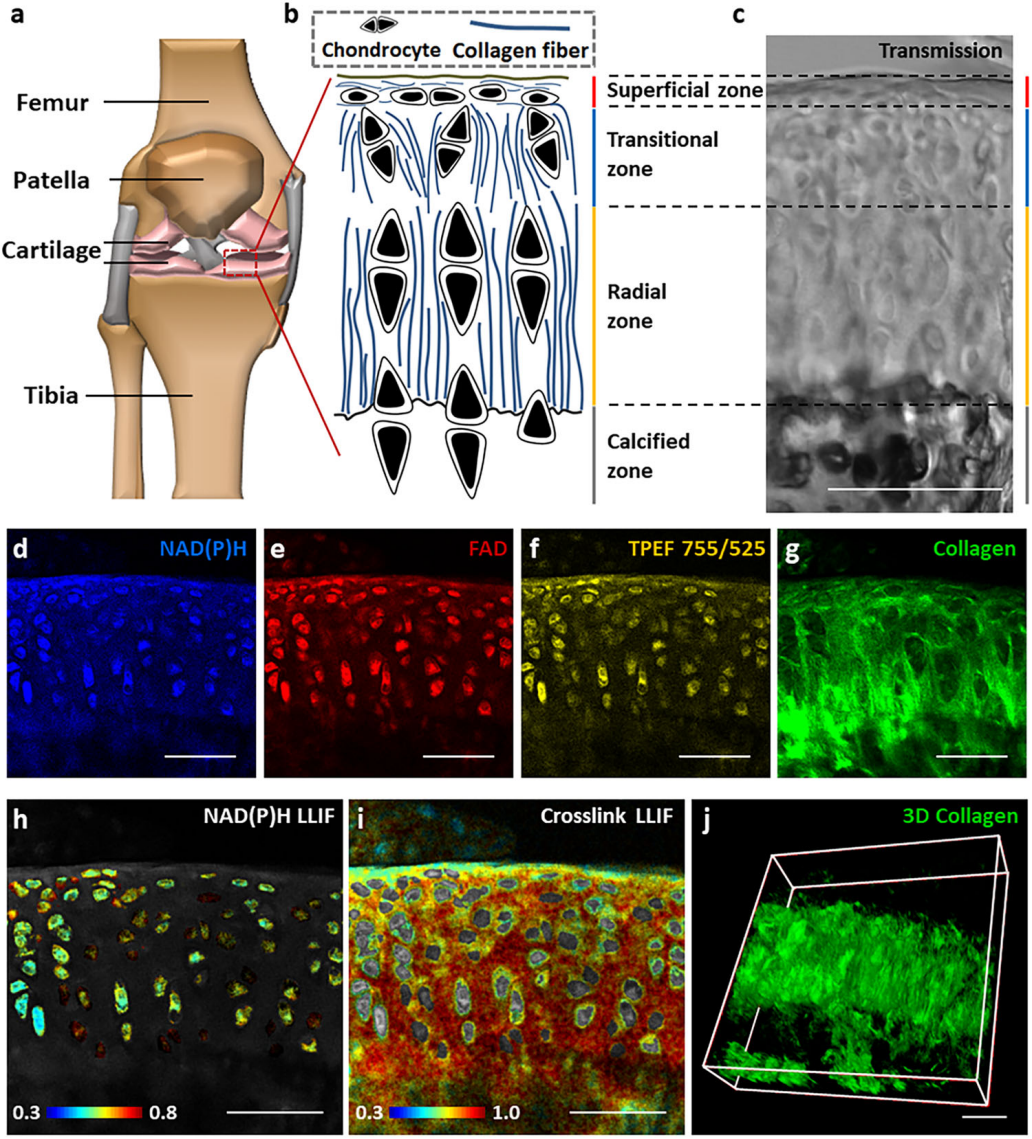
An overview of articular cartilage organization and different types of endogenous contrast for TPEF and SHG imaging. **a** Schematic (generated using Microsoft PowerPoint) showing the structure of bone, with the location of articular cartilage marked. **b** Schematic showing the composition of cartilage, which can be distinguished into four different zones: superficial, transitional, radial and calcified zone. **c** A representative transmission image from a mouse knee cryosection showing the segmentation of these four zones. Representative **d** NAD(P)H (755 nm ex./460 ± 20 nm em.), **e** FAD (860 nm ex./525±25 nm em.), **f** TPEF signal at 755 nm ex./525 nm em., **g** SHG image (920 nm ex./460 ± 20 nm em.), **h** NAD(P)H LLIF-coded image, and **i** cross-link LLIF-coded image from the same field. **j** A representative 3D SHG image stack used for the assessment of collagen fiber organization in a 3D context. Scale bar: 50 μm.

Articular cartilage is hyaline cartilage on the articular surfaces of bones, and lays inside the cavity of synovial joints (Fig. 1a). It is a typical biological tissue that includes chondrocytes and collagen fibers of distinct orientations depending on the location within the tissue. As shown in the schematic diagram (Fig. 1b) and a representative transmission image (Fig. 1c) of mouse articular cartilage, this tissue is divided into four zones: superficial, transitional, radial, and calcified^26^. The chondrocytes and collagen fibers are oriented parallel to the articular surface in the superficial zone, while perpendicularly to this surface in the radial zone. In the transitional zone, the chondrocytes are oriented at an angle to the surface, surrounded by territorial matrix composed of sheets of collagen fibers, which might lead to extra randomness^26^. As expected, our previous assessments demonstrated that the collagen fibers within the transitional zone were not aligned as well as those within the superficial or the radial zone^27^. These characteristics regarding the alignment of chondrocytes and collagen fibers guided us to segment each image into these distinct zones, which enabled us to assess functional and structural properties separately within each zone (see Methods). To assess the redox metabolic status and collagen fiber landscape in the articular cartilage, we acquired TPEF and SHG images from both cells and ECM for high-resolution, quantitative assessments. Representative NAD(P)H, FAD and collagen cross-link TPEF images are shown along with a collagen fiber SHG image (Fig. 1d–g, and Supplementary Video 1). The corresponding NAD(P)H (Fig. 1h) and collagen cross-link (Fig. 1i) LLIF-coded images were prepared using phasor analysis of the associated FLIM images. A reconstruction of a 3D SHG image stack is also included (Fig. 1j), as this was the type of data we used to assess 3D fiber orientation.

### Significant matrix remodeling can be detected as early as one week post OA-inducing surgery

Using the 3D SHG stacks and an approach we described in detail previously, we determined the azimuthal, $\theta$, and polar, $\varphi$, angle of fibers within each voxel (Supplementary Fig. 1a, b). Collagen fibers within the superficial zone were oriented primarily along either 0 or 180 degrees (i.e., parallel to the articular surface), while those in the radial zone were aligned along 90 degrees (i.e., perpendicular to the surface); the distributions of both $\theta$ and $\varphi$ angles were broader in the transitional zone than the other two zones, consistent with the expected organization^28^. The 3D directional variance was calculated from these angular distributions and corresponding maps with values that always varied between 0 (fully aligned) and 1 (completely random) were generated with dark hues indicating a more disorganized structure (Supplementary Fig. 1c, d).

**Fig. 2:**
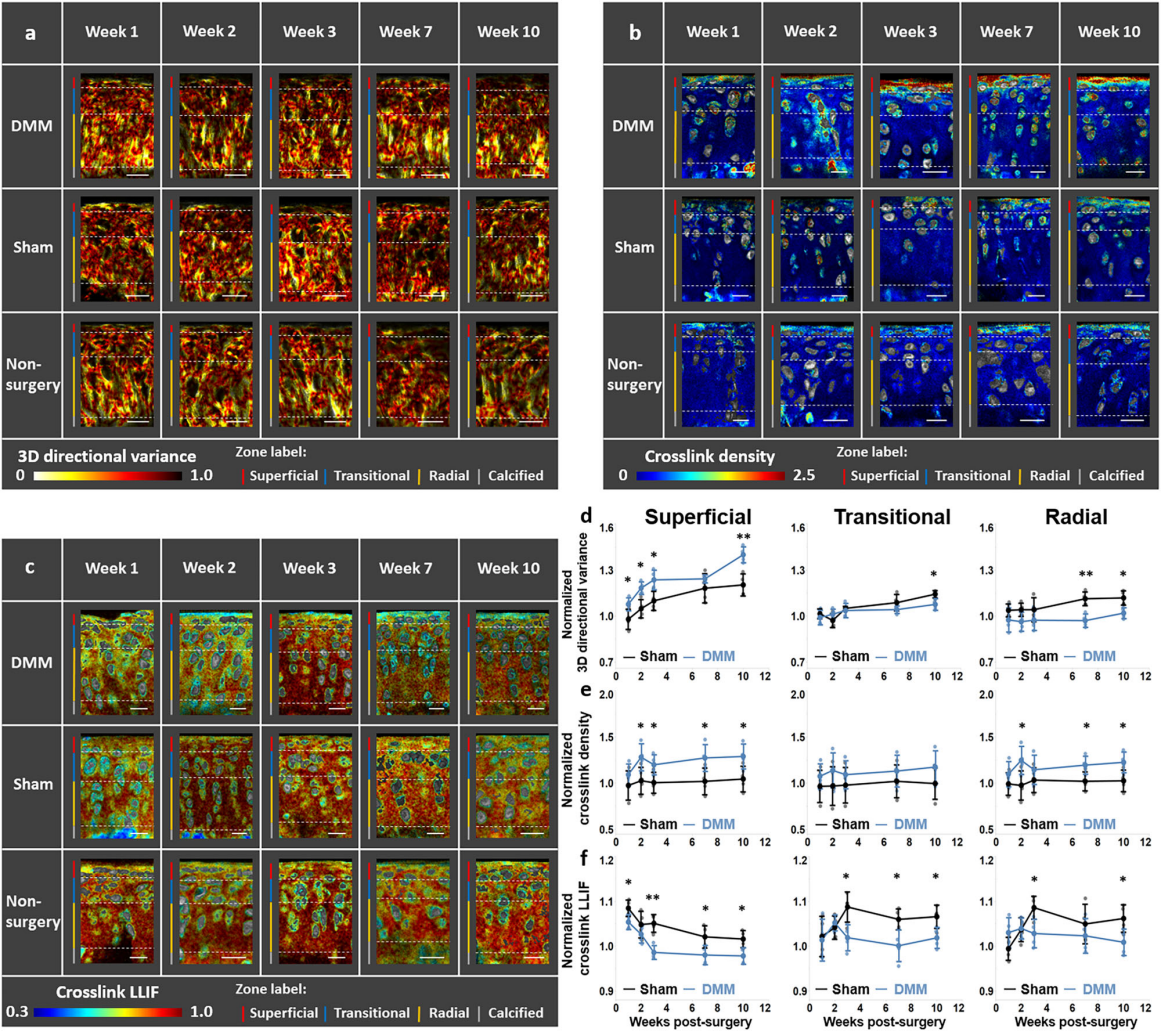
Optical metrics acquired from ECM label-free imaging at early stages of osteoarthritis. Representative maps of **a** 3D directional variance, **b** cross-link density, and **c** cross-link LLIF of the DMM (top), sham (middle), and non-surgery control (bottom), at 1, 2, 3, 7, and 10 weeks post-treatment. Different zone regions are indicated by different colors on the line adjacent to the specimens. The mean and standard deviation of **d** 3D directional variance, **e** cross-link density, and **f** cross-link LLIF normalized to non-surgery controls at different time points and distinct zones. *p < 0.05 and **p < 0.01. *n* = 4 mice for each group. Scale bar: 30 μm.

Such 3D variance coded maps are shown for representative images acquired from knee cryosections of BALB/c mice at 1, 2, 3, 7, or 10 weeks post DMM or sham surgery (Fig. 2a). Non-surgery controls were also imaged and used to normalize the DMM and sham surgery specimen results. Images were acquired from four mice from each group. Distinct zones in each map are labeled by different colors of the vertical side bar and segmented by a white dashed line; therefore, the hues in each zone can be directly compared to yield a qualitative impression of the fiber organization. Although subtle, these maps revealed that following DMM surgery, collagen fibers within the superficial zone typically yielded darker hues, indicating a potential disruption in fiber organization at the onset of OA (Fig. 2a). To represent these results quantitatively, the 3D directional variance value corresponding to each zone was acquired by averaging the values of all voxels within a zone, with the mean and standard deviation from all samples shown in Fig. 2d. Note that all the data were normalized to the non-surgery control specimen values, and the asterisks indicate whether there was a significant difference between DMM and sham at each specific time point. Consistent with hues shown in the 3D directional variance maps (Fig. 2a), the collagen fibers in the superficial zone under DMM treatment exhibited a significantly higher variance level, corresponding to a degradation in alignment, almost for all the time points except week 7. As a highly-sensitive tool, this 3D analysis was able to identify significant differences as early as week 1. It is interesting to observe that the collagen fiber organization changes followed an opposite trend in the transitional and radial zones compared to those in the superficial zone, with DMM leading to lower 3D variance, i.e., higher levels of collagen fiber alignment, especially at later time points.

The segmentation of these different zones was validated by polarized imaging of picrosirius red-stained sections, because picrosirius bound to collagen and displayed birefringence, allowing us to visualize collagen fibers (Supplementary Fig. 2). Using representative 10-week samples, we acquired sequentially polarized and SHG images of the same field. The zone borders indicated by the collagen fiber orientation and corresponding 3D directional variance values generated from SHG images, were highly consistent with those identified by polarized imaging. Moreover, our findings regarding changes in the 3D organization of collagen fibers within the DMM-treated samples were consistent with changes we observed in fixed and paraffin-embedded sections from articular cartilage samples from mice treated with monosodium iodoacetate (MIA, a spontaneous chemically induced OA model)^29^. Specifically, histological staining (Supplementary Fig. 3) and associated SHG and 3D directional variance maps (Supplementary Fig. 4) highlighted collagen loss and degradation of fiber organization within the superficial and radial zones of the MIA-treated samples compared to the control (PBS-treated) ones.

Representative collagen-associated TPEF images at 755 nm excitation/525 nm emission, correlating directly with collagen cross-link levels in our previous studies^25,30^, are shown in Fig. 2b. We note that the intensities of the images included in Fig. 2b were normalized to the corresponding SHG intensity values, so that they represented cross-link density. The unnormalized intensities are included in Supplementary Fig. 5. Interestingly, while the overall levels of cross-link-associated fluorescence decreased in the superficial zone of the DMM specimens, the cross-link density increased significantly as early as two weeks after surgery and remained elevated (Fig. 2e). Significant consistent enhancements in cross-link fluorescence density were observed in the radial zone at the 7 and 10 week time points (Fig. 2e), similar with the timeline of the appearance of fiber re-organization changes in this zone (Fig. 2d). Corresponding fluorescence lifetime maps of collagen cross-links at different zones and time points, represented by the cross-link LLIF, are shown in Fig. 2c. The phasors representing the SHG positive/collagen-rich FLIM image regions had a highly elliptical shape (Supplementary Fig. 6a), consistent with a bi-exponential decay. When a line was fitted to such an ellipse, it intersected the universal semicircle of the phasor space at points that represented the short- and long-lifetime components, with future bio-assays needed to resolve what these components were. The location of the phasor of each pixel along this line represented the LLIF, which always ranged between 0 and 1 (Supplementary Fig. 6a), with a higher/lower LLIF indicating more/less contributions from the long-lifetime component and corresponding to a longer/shorter lifetime. Changes in parameters of ECM, such as pH, viscosity, and oxygen content, might alter the exact lifetime of the long/short-lifetime components and/or their relative contributions^31^, making cross-link lifetime measurement a potential tool for evaluation of the milieu of chondrocytes. The superficial zone of the DMM samples typically exhibited bluer LLIF hues than the sham group, indicating a relative enhancement in the levels of cross-links with a shorter lifetime. These qualitative observations were confirmed by quantitative analysis of the images (Fig. 2f). The LLIF was consistently significantly lower within the superficial zone of the DMM samples (Fig. 2f). However, consistent and significant differences in the cross-link LLIF were observed in the transitional zone as early as week 3 (Fig. 2f). Collectively, these studies highlighted that label-free, two-photon imaging revealed significant changes in the collagen fiber structural and chemical integrity within the superficial zone of cartilage tissues immediately upon the onset of OA (within the first week of DMM surgery). Changes in the radial zone were detected with some delay (seven weeks post DMM surgery), but before the onset of pain (expected at ~week 10 or later)^32^. Thus, the high spatial resolution conferred by these microscopic measurements was critical for detecting such heterogeneous responses in a sensitive manner. In addition, our results revealed that collagen fiber organization and content as well as the nature of the cross-links within the cartilage matrix were all relevant and significant contributors to OA-associated matrix remodeling.

### Chondrocytes undergo dynamic metabolic reprogramming at early stages post OA-inducing surgery

**Fig. 3:**
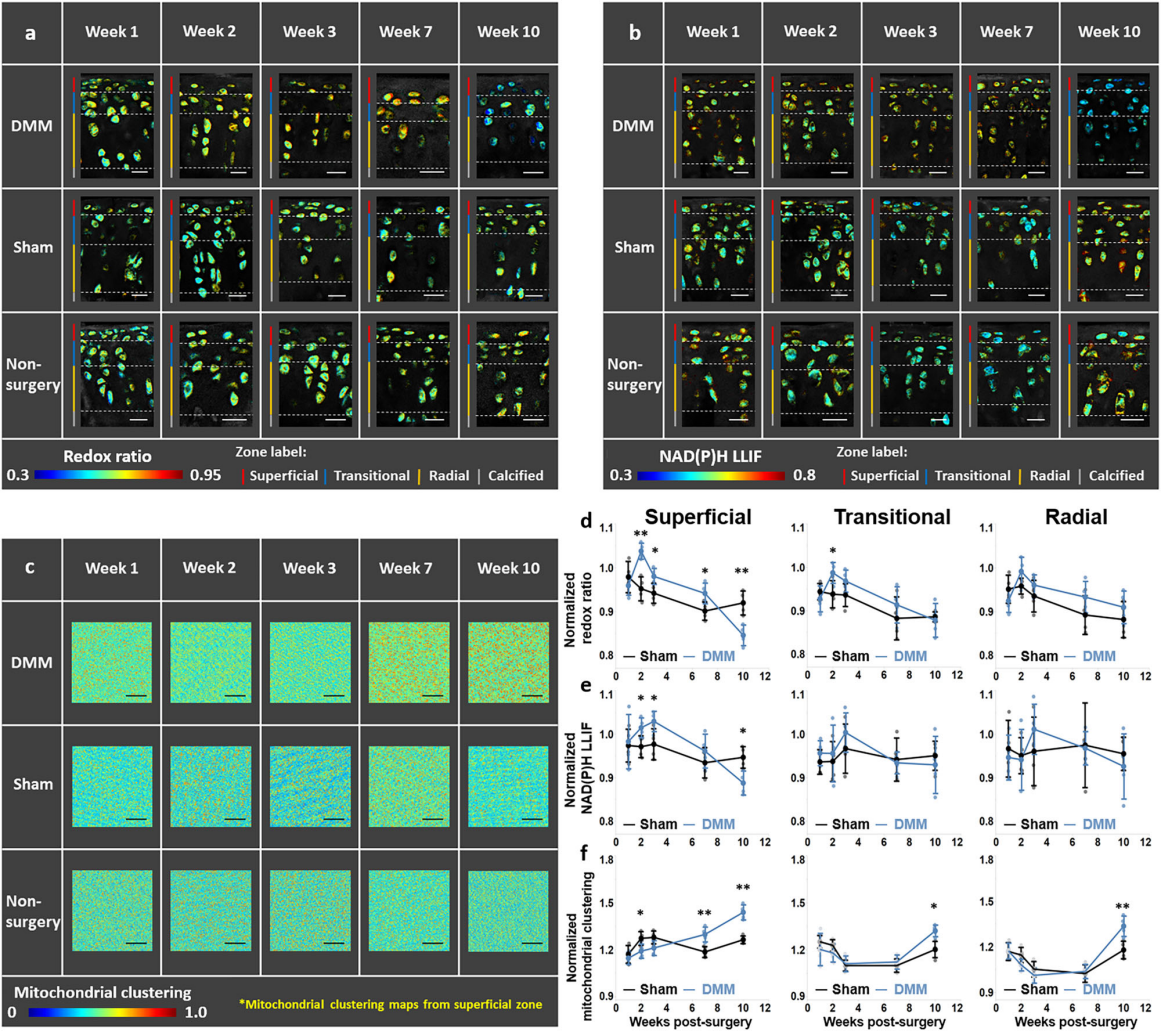
Cellular metabolism changes dynamically at early stages of osteoarthritis. Representative maps of **a** redox ratio, **b** NAD(P)H LLIF, and **c** mitochondrial clustering of the DMM (top), sham (middle), and non-surgery control (bottom), at 1, 2, 3, 7 and 10 weeks post-treatment. The mean and standard deviation of **d** redox ratio, **e** NAD(P)H LLIF, and **f** mitochondrial clustering normalized to non-surgery control at different time points and distinct zones. *p < 0.05 and **p < 0.01. *n* = 4 mice for each group. Scale bar in **a** and **b**: 30 μm; Scale bar in **c**: 100 μm.

Focusing on the cellular components of imaged fields, we sought to exploit the label-free intensity and lifetime TPEF images to assess the presence and dynamics of OA-associated metabolic reprogramming. We extracted several optical metrics of metabolic activity. The optical redox ratio, defined by our group and others as the fluorescence intensity ratio of: FAD/[NAD(P)H + FAD], was acquired from the corresponding FAD and NAD(P)H intensity images on a per pixel basis, and always varied between 0 and 1 (Supplementary Fig. 7). Representative redox-ratio coded images are included in Fig. 3a, highlighting subtle heterogeneities in the metabolic function of cells within a given field, and more obvious hue changes over time in the DMM samples compared to the sham and non-surgery control ones. These qualitative features were also detected within NAD(P)H LLIF-coded images (Fig. 3b). Clone-stamped NAD(P)H intensity images from the superficial zone of the specimens we imaged are included in Fig. 3c. While more difficult to interpret intuitively, they indicated differences in the frequency patterns of the intensity fluctuations (Supplementary Fig. 8), which were analyzed to yield insights on mitochondrial organization changes. The patches of higher intensity (yellow/red hues), such as the ones observed from DMM specimens at the 7 and 10 week time points, corresponded to higher levels of mitochondrial clustering or fragmentation. In contrast, relatively uniform intensity distributions corresponded to lower levels of clustering and more highly networked mitochondria. In agreement with the matrix results, we detected significant changes primarily within the superficial zone of the cartilage as early as two weeks following surgery (Fig. 3d–f). However, the relative increase in redox ratio and NAD(P)H LLIF and decrease in mitochondrial fragmentation observed in the DMM samples at the 2 week time point, were reversed at seven and ten weeks following surgery (Fig. 3d–f). Together, these results revealed dynamic metabolic reprogramming of the superficial zone cells. Changes in mitochondrial organization were detected within the transitional and radial zones at the 10 week time point and represented OA-associated metabolic reprogramming in a highly sensitive manner more broadly. Since collagen fiber changes were detected as early as one week, our findings indicated that metabolic changes followed modifications in the surrounding matrix.

### Human OA specimens exhibit cell metabolic and matrix changes similar to the ones observed within the mouse OA model specimens

**Fig. 4:**
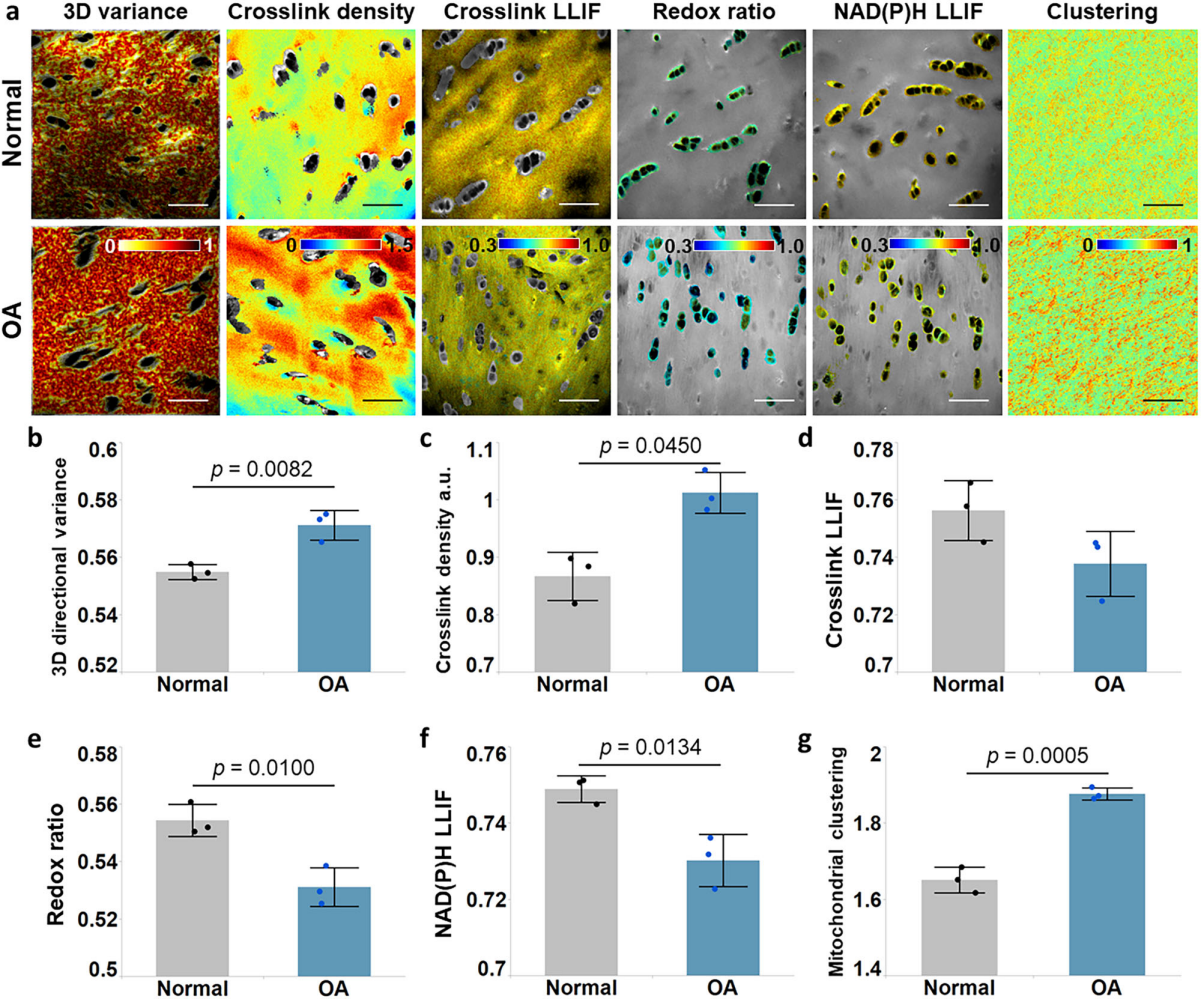
Measures of 3D collagen fiber organization, cross-link degradation, and cellular metabolism in human articular cartilage suffering from OA. **a** Representative maps of (from left to right): 3D directional variance, cross-link density, cross-link LLIF, redox ratio, NAD(P)H LLIF and mitochondrial clustering, obtained from normal (top) or OA samples (bottom). Patient-based mean and standard deviation of **b** 3D directional variance, **c** cross-link density, **d** cross-link LLIF, **e** redox ratio, **f** NAD(P)H LLIF, and **g** mitochondrial clustering, with *p* values indicated in each graph. *n* = 3 patients for each group. Scale bar: 50 µm.

To assess the possible relevance of our findings in the mouse DMM OA model to OA in humans, we imaged and analyzed cartilage specimens from human subjects suffering from OA. Specifically, articular cartilage specimens were harvested from knee joints of three patients with OA, who were 63, 65, and 81 years old. Cartilage from the unaffected region served as normal controls. These unaffected and affected regions were validated by Safranin O/Fast Green staining and imaging, with affected regions exhibiting obvious loss of glycosaminoglycan (GAG) (Supplementary Fig. 9). Sections were made parallel to the surface of the articular cartilage. Because the ages of the donors were generally advanced, cartilage samples tended to be less intact, and could expose cartilage beneath the superficial zone in some areas. Figure 4a shows representative maps of the collagen fiber organization, collagen cross-link properties, and cellular metabolism from the OA-affected and presumably “normal” (non-OA affected) tissue sections. Differences in the hues of each one of these metrics between OA and “normal” samples were visible (Fig. 4a), and quantified for all samples (Fig. 4b–g). Except for the cross-link LLIF (p = 0.067), all the other optical metrics exhibited significant differences between the OA and “normal” groups. Interestingly, the differences between OA and normal human samples followed the trends we observed between the DMM and sham mouse knee samples at week 10, confirming the potential relevance of the model and the label-free optical metrics we reported.

### A combination of cellular optical biomarkers provides insights into the nature of metabolic reprogramming

**Fig. 5:**
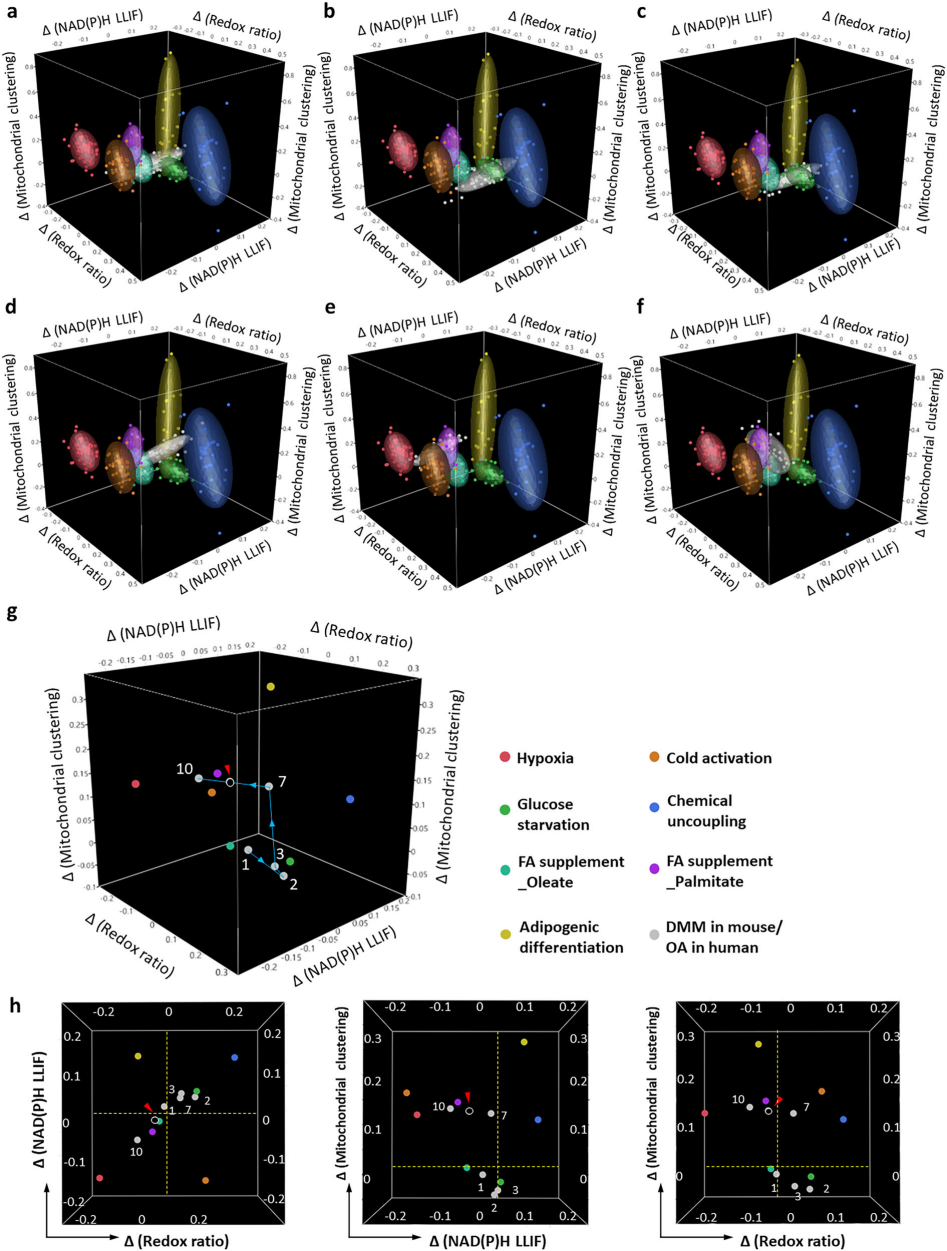
Insights on OA-associated metabolic reprogramming using previously established optical metabolic pathway signatures. Combined optical metabolic readout changes have been reported from similar label-free two-photon images of cells exposed to specific metabolic perturbations including^19^: hypoxia (enhanced glycolysis), glucose starvation (enhanced glutaminolysis/oxidative phosphorylation), chemically (CCCP)- and physiologically (cold activation)-induced uncoupling, unsaturated (oleate) and saturated (palmitate) fatty acid supplementation (oxidation), and induction of adipogenic differentiation (fatty acid synthesis). The three axes correspond to changes (relative to corresponding controls) of the redox ratio, NAD(P)H LLIF, and mitochondrial clustering. The ellipsoid surfaces incorporate 75% of all the data for a particular metabolic perturbation. In the same parameter space, we include the field-based DMM data (relative to sham surgery controls) at **a** Week 1, **b** Week 2, **c** Week 3, **d** Week 7, and **e** Week 10, and **f** the human OA (relative to healthy controls) specimen changes. **g** Optical metabolic scatterplot, with each metabolic perturbation represented by the centroid of each population. The DMM data points for each week are color-coded by gray hues and each distinct time point is marked by number. The human OA data point is indicated by the red arrow. The blue line indicates the change of cellular metabolism from Week 1 to Week 10. The hue labels for different metabolic perturbations to the right of the 3D scatterplot apply for all the figure panels. **h** Corresponding 2D projections of the 3D scatterplot in (**g**). The dashed yellow lines indicate the zero level of each metric.

In order to gain insights regarding the nature of the optical metabolic changes we observed in response to OA pathogenesis, we mapped the optical redox ratio, NAD(P)H LLIF, and mitochondrial clustering changes of DMM (or OA) relative to sham (or normal) into our previously validated database of cellular optical metabolic changes observed in response to perturbations in distinct metabolic pathways, including glycolysis and glutaminolysis, extrinsic and intrinsic mitochondrial uncoupling, and fatty acid (saturated or unsaturated) oxidation and synthesis (Fig. 5 and Supplementary Video 2)^19^. The range of cellular optical metabolic changes we detected corresponding to each perturbation was represented by ellipsoids of different hues, while the changes detected in our DMM-injured (human OA) samples relative to the corresponding sham (human “normal”) samples were gray coded (Fig. 5a–f). To visualize these alterations more readily, we included the centroid of each ellipsoid in Fig. 5g, and the corresponding projections along one of the three axes (Fig. 5h). The numbers indicated the corresponding DMM treatment time points while the human OA data was marked by a red arrow in the 3D scatterplot and the two-dimensional (2D) projections. The changes observed at week 2 and 3 were consistent with the changes we observed in our previous studies upon glucose starvation, which led to enhanced glutaminolysis and oxidative phosphorylation. At the later time points, the observed DMM-associated metabolic changes were in the vicinity of changes we detected upon enhanced glycolysis (in response to hypoxia) and saturated fatty acid oxidation (in response to palmitate supplementation).

### Informational complementarity from functional and structural features leads to insights into the presence of OA-associated changes and the time-dependent progression of OA pathogenesis

**Fig. 6:**
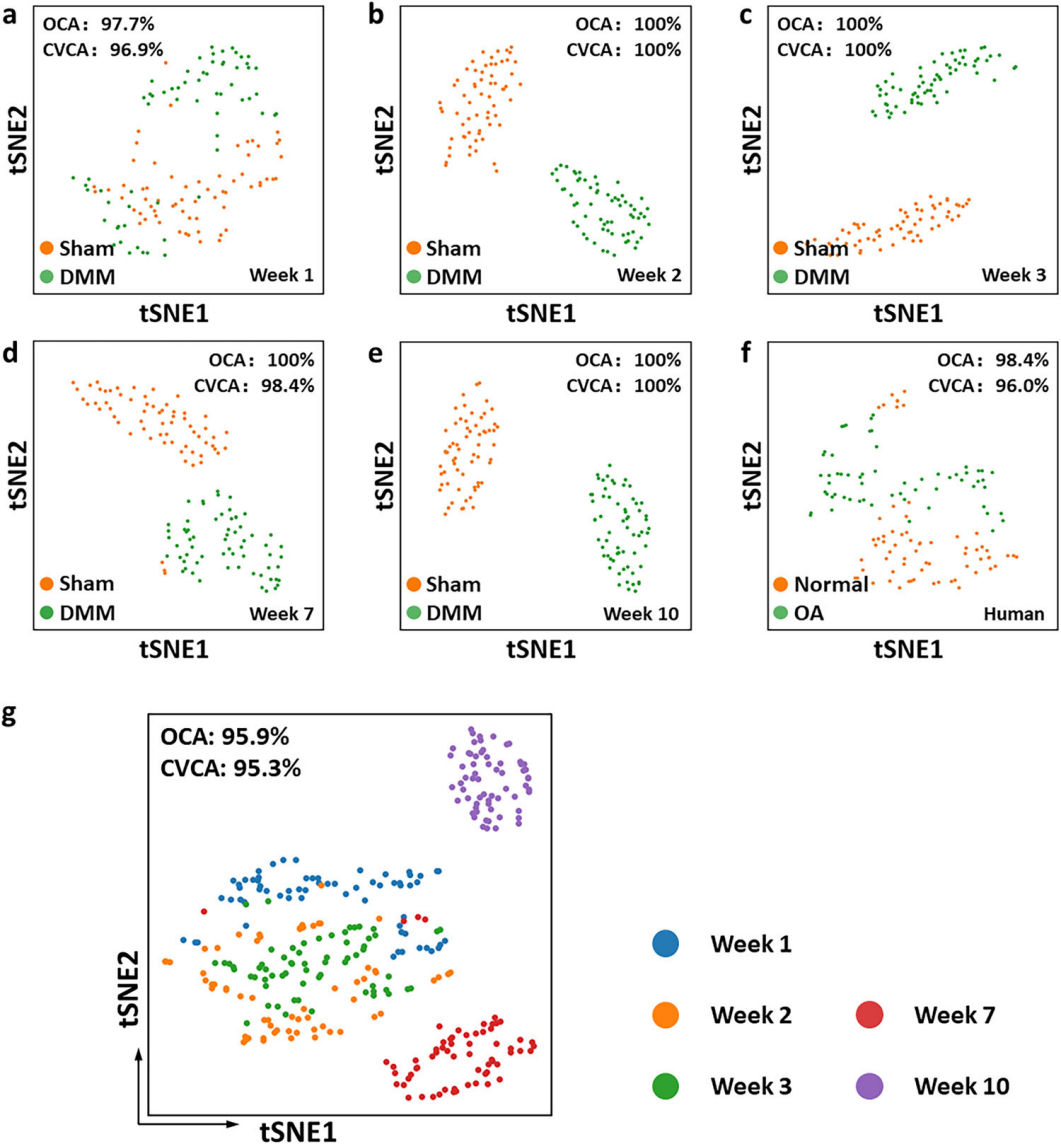
viSNE-based visualization within and between group separation based on the combination of the six optical metrics. viSNE maps of mouse model data at **a** Week 1, **b** Week 2, **c** Week 3, **d** Week 7, and **e** Week 10, and **f** human specimen data. **g** Overall viSNE map showing the clustering of field-based data in response to DMM surgery at different time points in the mouse model. DMM data are normalized to the corresponding optical metric mean value from the sham group at each time point. Different colors correspond to different time points, as labeled to the right of the map. OCA and CVCA values are marked in each viSNE map.

Overall, we obtained six optical metrics corresponding to functional and structural features of articular cartilage, from both cells and ECM. Multicollinearity diagnostics was performed for mouse data from distinct time points and human data, and supported the lack of multicollinearity (Pearson product-moment correlation coefficients, $|r| < 0.7$, with representative results from week 10 mouse data shown in Supplementary Fig. 10), revealing that there were no offending variables that needed to be eliminated and the independence of these variables. In order to further investigate the potential impact of the informational complementarity of these optical biomarkers to identify the onset of OA, we performed linear discriminant analysis using the data from the superficial zone and assessed the overall and cross-validated classification accuracy (OCA and CVCA, respectively) of the model (Table 1). The number of independent samples for this analysis was small, but the superb classification accuracy of the model highlighted the sensitivity with which these readouts reported on OA-associated changes from a very early stage. This high discriminatory power of the combined metrics was highlighted (Fig. 6a–f) via viSNE analysis, which is a statistical dimension-reduction technique that enabled visualization of the clustering of features attributed to a given group at distinct time points or from different specimens by considering contributions from these six biomarkers. The sham and DMM specimens became increasingly distinct over three weeks. While the separation was most obvious at the 10 week time point, this was preceded by a period (7 week time point) of significant remodeling (especially in terms of metabolism) when metrics from the two groups appeared closer and yielded less than perfect discrimination. The classification importance of each metric is reported in Supplementary Table 1. The viSNE plots included in Supplementary Figs. 11–16 provide a visual assessment of the variation of each metric across the DMM and sham specimens over time as well as between the “normal” and OA human specimens. The progression of changes as a function of time for the DMM specimens is highlighted in Fig. 6g. The six-metric combination quantitatively yields 95.9% OCA and 95.3% CVCA for classifying the 320 fields into the five time points examined (64 image fields from each time point, as detailed in “Methods”). We note that these are DMM data normalized to the corresponding mean sham levels at each distinct time point. These studies collectively demonstrate the potential of using a combination of endogenous optical readouts to acquire sensitive and quantitative insights into not only the presence of OA-associated structural and/or functional changes but also the time-dependent progression of OA pathogenesis in terms of the interplay of matrix and cellular metabolic remodeling.

**Table 1:** Classification accuracy between DMM (OA) and sham (normal) using a single optical parameter.

Parameter^a^							Classification accuracy					
1	2	3	4	5	6		Week 1	Week 2	Week 3	Week 7	Week 10	Human
●						OCA	74.2%	89.1%	92.2%	64.1%	94.5%	64.3%
						CVCA	74.2%	88.3%	92.2%	64.1%	94.5%	64.3%
	●					OCA	68.0%	75.8%	82.8%	82.0%	76.6%	74.6%
						CVCA	68.0%	75.0%	82.0%	82.0%	75.8%	74.6%
		●				OCA	64.8%	57.0%	84.4%	73.4%	70.3%	66.7%
						CVCA	64.8%	56.3%	84.4%	73.4%	70.3%	66.7%
			●			OCA	56.3%	86.7%	69.5%	67.2%	89.8%	64.3%
						CVCA	56.3%	85.9%	69.5%	67.2%	89.8%	64.3%
				●		OCA	53.1%	68.8%	71.9%	64.1%	84.4%	71.4%
						CVCA	53.1%	68.8%	71.9%	64.1%	84.4%	71.4%
					●	OCA	56.3%	65.6%	71.9%	91.4%	89.8%	81.7%
						CVCA	56.3%	64.8%	71.9%	91.4%	89.8%	81.7%

^a^Each number corresponds to a specific optical parameter, detailed as follows.

1: 3D directional variance; 2: cross-link density; 3: cross-link LLIF; 4: Redox ratio; 5: NAD(P)H LLIF; 6: Mitochondrial clustering.

## Discussion

**Fig. 7:**
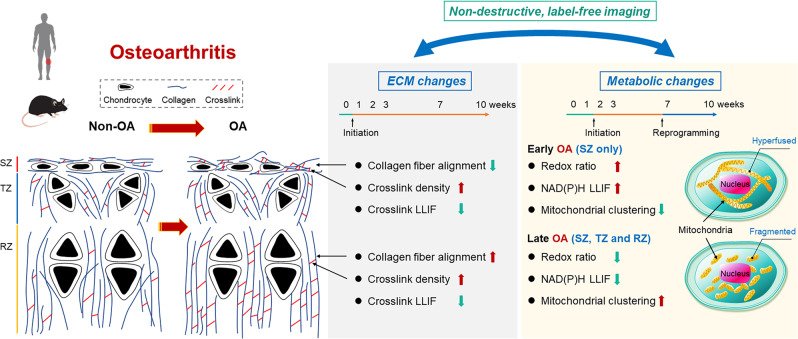
Schematic of matrix and cell metabolic changes at early stages of osteoarthritis. SZ: Superficial zone; TZ: Transitional zone; RZ: Radial zone. This figure, including every element, is generated using Microsoft PowerPoint.

In this study, we report on dynamic matrix remodeling and metabolic reprogramming that occur upon the onset of osteoarthritis. Our primary evaluations are performed with cryosections from mouse knees at various points from one to ten weeks following DMM (OA-inducing) or sham surgery. These times represent the very onset of the disease, since OA-associated pain typically develops ten weeks following surgery. A main innovative feature of our study in the context of OA is the use of label-free, high-resolution, multi-modal two-photon imaging to assess at the same time the state of cellular function and matrix integrity. As anticipated, changes are primarily detected in the superficial zone, as shown in the schematic model that summarizes the structural and functional changes at early OA stages reported in this study (Fig. 7), highlighting the importance of spatial resolution. We identify significant changes as early as the first week following DMM surgery in the content and organization of the collagen fibers, along with the fluorescence lifetime of collagen cross-links. It is worth mentioning that the high-resolution imaging and quantitative characterization enable more sensitive identification of organizational remodeling at the onset of OA than traditional methodologies, such as Safranin O/Fast Green staining, which validates that early OA takes place following DMM surgery. However, note that staining identifies differences between DMM and OA starting from week 7 only (with representative images shown in Supplementary Fig. 17), as was the case from our prior study^33^. Safranin O staining was quantified using an established grading method^34^ where a score was given based on the percentage of matrix loss (below 1/3, 1/3–2/3 or over 2/3 in depth and surface area), with the highest score being 12 and lowest score being 0. Functional metabolic changes are detected during the second-week time point; however, the direction and level of changes in the optical metabolic readouts we characterize indicate a significant shift in the nature of the metabolic pathways that are utilized between three and seven weeks. Interestingly, at three and seven weeks we also detect significant changes in some or all of the matrix associated metrics of the transitional and radial zones. It is also of note that for the first and third-week time points all three of the most discriminatory variables for the DMM-associated changes are matrix associated, while for all other groups a combination of metabolic and matrix associated readouts is most relevant (Supplementary Table 1). Thus, our data reveal a rather dynamic interplay between matrix and cellular functional changes and the unique capabilities of our approach to characterize these changes in the same specimen.

Throughout this study, both mouse and human cartilage specimens underwent sucrose equilibration and cryosectioning. In order to ensure that these processing treatments did not introduce artifacts on intrinsic signals of the ECM and cells leading to observation of erroneous differences, we compared the fluorescence and SHG readouts from frozen and sucrose equilibrated and cryosectioned porcine cartilage tissues (for ECM assessments, Supplementary Figs. 18 and 19) and mouse epithelial tissues (for metabolic assessments, Supplementary Fig. 20). As can be seen from representative images and corresponding metrics, while the absolute values of metrics may have been impacted by the processing, differences in all metrics between control and trypsin treated cartilage tissues as well as superficial and deeper epithelial tissue layers were very similar among frozen and sucrose equilibrated/cryosectioned tissues. Thus, we expect that the key differences we report in matrix and cell metabolic readouts between control and osteoarthritis-developing samples are not impacted by the mild sucrose fixation and cryosectioning. In addition, it is worth mentioning that other auto-fluorescent molecules are not likely to interfere with our measurements here. Keratin and melanin are not expected to be present in cartilage, and contributions that are likely associated with intensely fluorescent lipofuscin are eliminated by thresholding^35^. Moreover, histochemistry studies suggested that little elastin (the other main potential endogenous fluorophore within the matrix) was present in articular cartilage^36^.

One of the key metrics of 3D collagen organization we report is the 3D directional variance, as quantified from SHG images. SHG imaging has advantages for investigating cartilage organization^21,37^ surpassing some other imaging approaches, including optical coherence tomography^38^, atomic force microscopy^39^, magnetic resonance imaging^40^, Fourier transformed infrared imaging^41^, and quantitative polarized light microscopy^42^, since they are in need of exogenous labels, lack high resolution, and/or are limited to acquisition of 2D images only. Since SHG is specific to non-centrosymmetric structure, and collagen fibers are the main components with such microstructure in cartilage, it is reasonable to assume that collagen fibers are the main voxel elements by selecting SHG positive voxels. Changes in this 3D directional variance metric are detected at the earliest time point within the superficial zone and become significant for the radial and transitional zones at seven and ten weeks, respectively. The observed increase in 3D directional variance of the superficial zone within the DMM relative to the sham specimens at each time point is indicative of collagen fiber organization loss (Fig. 2). These observations are consistent with previous studies on human cartilage at early OA stages (OARSI grade = 1.0–1.5) using polarized light microscopy^5^ and on sheep cartilage 8 months post induction of medial meniscal tear using MRI^43^. Higher resolution imaging approaches, such as polarization sensitive optical coherence tomography, revealed similar changes in ex vivo unstained human articular cartilage sections of sub-millimeter thickness obtained either post-limb amputation or post-joint resection, using scores with a number ranging between 0 and 2 for quantification of collagen fiber disorganization based on the movement of polarization bands in the image^44^. To understand underlying molecular mechanisms of collagen degradation in OA, robust mass spectrometry imaging (MSI) work was employed to identify various disease-specific markers that differentiated OA and healthy cartilage in humans; potential OA markers, including cartilage oligomeric matrix protein (COMP) and fibronectin, were determined to be associated with structural alterations in ECM^45^. Significant GAG losses have also been reported at early stages of OA in human specimens within the superficial zone^46^, and these could certainly contribute to the observed changes in collagen organization since they are important supportive elements of the collagen fiber matrix^47^. The loss of GAG in OA is well-established, but the change of the collagen landscape is much less understood. Our study on 3D changes in OA provides key understanding on overall matrix organizational degradation, especially within the superficial zone where the destruction initiates. We note, that detection of collagen fiber organization changes as early as one week post the onset of DMM or related surgery has not been reported. In fact, in a previous study we performed using the same mouse OA model and 2D analysis of corresponding SHG images, we identified the earliest significant differences at week 3 post-surgery^33^. This enhanced sensitivity conferred by analysis of the 3D vs. the 2D collagen fiber organization characteristics is consistent with our findings in other disease models^27,48^.

Interestingly, in the transitional and radial zones, the collagen fibers become more organized following DMM surgery compared to sham-surgery, as early as 7 weeks post-surgery. It is not clear whether distinct cell-matrix interactions in these zones vs. the superficial zone may play a role in these differences or whether the distinct collagen organization patterns of normal cartilage in these zones are responsible for this response. However, this is an interesting finding that merits further investigation.

Changes in collagen fiber organization are followed by modifications in the collagen cross-linking, detectable at two weeks in the superficial zone and as early as two weeks in deeper zones as well. In this study, the TPEF signal at 755 nm excitation/525 nm emission was used to represent the cross-linking level, as verified by mass spectrometry-based assessments from collagen type I of tendon tissues^25^. In normal articular cartilage, 90% of the collagen content is type II collagen (Col II); however, other collagen isoforms (IX and XI) fibrils are co-mingled to form fibers dictating the tissue anisotropy of articular cartilage^21^. Although the collagen type is different in cartilage and tendon tissues, this mass spectrometry-validated method for quantifying cross-linking level is relevant because all the fibril-forming collagen types (types I, II, III, V, and XI) are cross-linked predominantly through the same mechanism based on the reactions of aldehydes generated enzymatically from lysine (or hydroxylysine) side-chains by lysyl oxidase^49^. While the overall cross-link content decreases during OA, as represented by the raw TPEF signal (Supplementary Fig. 5), the cross-link density of the fibers that remain in the cartilage increases significantly. Our results are consistent with the study by Manning et al. where a decrease in autofluorescence intensity (355 nm ex./410 ± 20 nm em.) was visualized within porcine articular cartilage including all zones degraded by treatment of matrix metalloproteinase 1 (MMP-1)^6^. Our observations are also consistent with results reported by Kim et al.^24^; they used immunostaining to characterize the levels of the collagen cross-linking enzyme lysyl oxidase (LOX) within cells, as a quantitative measure of matrix cross-linking level, and they observed an increase in LOX expression in OA-induced (DMM) mice specimens from week 1 to week 8 post-surgery^24^. We note, that while the fluorescence we detect is not directly attributed to LOX-mediated cross-links, we have found that this signal correlates highly with LOX-mediated cross-links and LOXL4 expression^25,50^. Changes in the cross-link LLIF are also detected early, in fact earlier than fluorescence cross-link-density differences (Fig. 2c, f). Our findings are consistent with a previous study which reported a lower autofluorescence lifetime from cross-links of degraded cartilage matrix by different proteinases (bacterial collagenase or MMP) using a compact multidimensional fluorometer^6^. These changes are likely attributed to a change in the balance of the nature of cross-links that are present. These organization changes in collagen fibers might also provide insights into the underlying mechanisms regarding biomechanical function changes at the superficial and partly transitional zones of murine femoral condyle cartilage, as represented by significantly reduced nanoindentation modulus 1 week post DMM surgery, observed from atomic force microscopy (AFM)-based nanoindentation^51^. These highly dynamic and spatially varying changes in collagen fiber organization and cross-linking indicate that the micromechanical environment experienced by chondrocytes is highly heterogeneous and more detailed understanding of these interactions is needed.

Thus, it is not surprising that the metabolic changes we observe within cartilage specimens are also highly dynamic and heterogeneous (Fig. 7). The elevated redox ratio and NAD(P)H LLIF and lower mitochondrial fragmentation observed two weeks following DMM surgery are consistent with enhanced oxidative phosphorylation, leading to abrogation of cytosolic, free NAD(P)H pools and more fused mitochondria to optimize energy delivery and possibly prevent mitochondrial autophagy^52,53^. Upregulation of oxidative phosphorylation has been reported in primary bovine chondrocytes in response to nutrient stress, and has been proposed as a means to promote cell survival^54^. Interestingly, at later time points and within human OA specimens, the observed optical metabolic readout changes indicate a reprogramming of metabolic function towards enhanced glycolysis and/or fatty acid oxidation. Previous observations support these findings. Cartilage from patients with OA at a later stage of disease has been shown to exhibit elevated glycolysis to maintain energy homeostasis^55,56^. Reactive oxygen species (ROS) involvement in OA pathogenesis has also been reported^57^. While the impact of ROS was not assessed in our previous study that characterized the impact of different metabolic perturbations on optical metabolic readouts, ROS have been shown to induce cell apoptosis, which was associated with an increase in redox ratio and NAD(P)H lifetime, thus partially balancing the decreased level of these two metrics related to enhanced glycolysis^58,59^. On the other hand, excess ROS production was found to cause depolarization and fragmentation of mitochondria^60^, which are also consistent with the changes in mitochondrial organization induced by enhanced glycolysis and saturated fatty acid synthesis (Fig. 5h). The observation of subcellular level metabolic changes and cell-to-cell heterogeneity is enabled by the high resolution offered by TPEF, which could not be achieved using traditional imaging tools, such as positron emission tomography (PET)^61^, magnetic resonance imaging (MRI)^62^, near-infrared (NIR) light-based imaging techniques^63–65^ and quantitative photoacoustic tomography (qPAT)^66–68^. Moreover, such assessments do not need to ionize the molecules of tissue samples, in contrast to techniques including mass spectrometry^69^ and MSI^70–72^ which enable detailed investigations into the spatial distribution of molecular species.

Prior studies have highlighted the importance and dynamic nature of the interactions between cellular metabolism and ECM in osteoarthritis^73^. Inflammation is known to cause increased glycolysis^74,75^. One way that increased glycolysis accelerates matrix damage is through the generation of a more acidic environment, which inhibits matrix synthesis^76^. Another way that increased glycolysis can promote matrix alteration is the induction of advanced glycation products (AGE)^77^. Indeed, elevated AGEs have been found in both human and experimental OA^78^, although future work is needed to validate changes of AGEs in our OA model. The increase in AGE results in an increase in collagen cross-linking^77^, which is consistent with our observations. Furthermore, AGE also can induce oxygen stress that further exacerbates inflammation, mitochondrial dysfunction, and chondrocyte cell death^79–81^. In this way, altered ECM in turn changes chondrocyte metabolism in OA.

In conclusion, our findings show that multi-parametric TPEF and SHG imaging of both cells and ECM relying on endogenous contrast is a valuable investigational approach that enables non-destructive and quantitative assessments of functional and structural alterations at an early stage of OA. Quantitative assessments of cells and ECM simultaneously provide not only complementary information regarding OA pathogenesis, but also insights regarding significant interactions between these tissue compartments. These interactions appear to be complex and dynamic and multiple techniques will be likely needed to gain a thorough understanding of the disease. Studies like ours can serve as a useful guide for more detailed genomic and proteomic studies, such as MSI, that define underlying biological mechanisms that produce these optical changes and might enable discovery of novel OA-specific analytes^82^. In order to indicate the translational potential of the optical characterizations we perform in the mouse DMM model to human OA, we quantify the same optical metrics from human specimens with advanced OA. We note that the analysis of human samples is included only to highlight the relevance of the metrics we describe and to motivate future studies. Unfortunately, OA disease is more highly advanced in the human specimens that are readily accessible, since in principle at early OA stage patients would not experience pain nor have any invasive procedures. It is also worth mentioning that DMM is just one of the OA models used for pharmacologic studies. However, the fact that our findings regarding the nature of matrix and metabolic changes in the mouse DMM model are consistent with differences we identify in human OA specimens is promising. Clearly, more extensive studies with other representative animal OA models and larger numbers of human specimens are needed to correlate more rigorously our findings with human OA disease. In this study, we were limited to assessments of cryosections, instead of in vivo metabolic and matrix remodeling analysis. Such measurements would require a probe, since two-photon imaging is limited to penetration depths that extend typically a few hundred microns from the tissue surface. However, recent developments of multi-photon microendoscopes may enable translation of such studies in vivo via minimally invasive procedures^83^. Such measurements will offer a unique potential to monitor OA development and the impact of novel treatments that aim to interfere with disease onset and progression instead of symptom relief.

## Methods

### Articular cartilage tissue preparation

A widely used OA-induction surgery, DMM, was performed on 8-week-old male BALB/c mice (Taconic, NY, USA) according to an established protocol^84,85^. Specifically, the right knee joint was opened along the medial border of the patellar ligament and the medial meniscotibial ligament was severed. The left knee joint received a sham surgery, in which the ligament was exposed, while not severed. The knee joints from mice undergoing no surgery served as non-surgery controls. We collected knee joints at 1, 2, 3, 7, and 10 weeks post-surgery, from four mice at each time point. These time points represented early pathological OA changes using this DMM model, in which pain typically developed at 10 weeks post-surgery. Since both metabolic functions and collagen organization would be compared between DMM and sham specimens, we pre-processed these samples by cryosectioning to guarantee the same thickness of all the samples to minimize the artifacts originating from different sample sizes. Specifically, instead of paraformaldehyde fixation, knee joint samples were equilibrated in a 30% sucrose solution for 3 days at 4 °C before being embedded in optimal cutting temperature (OCT) compound. Sucrose was a common cryoprotectant for cryopreserving, used to prevent the formation of ice crystals, which may compromise the integrity of the cell membrane, produce holes within cells and loosen the ECM^86^. OCT-embedded joints were cryosectioned sagittally at 40 µm thickness. Mouse joint slices mainly included superficial, transitional, and radial zones of cartilage from the tibia, but also some other regions from the tibia and/or the femur. Only the tibia regions were considered for our study. All animal procedures were approved by the Tufts University Institutional Animal Care and Use Committee (IACUC), and we complied with all relevant ethical regulations for animal testing and research.

The segmentation of different zones was validated by picrosirius red staining and polarization-sensitive imaging. Specifically, picrosirius red staining was performed on mouse cartilage samples at 10 weeks post-surgery following a standard protocol, in which sections were stained by 0.1% Sirius Red in saturated picric acid for 1 hour at room temperature, followed by washing with deionized water^87^.

To compare our OA-associated readouts with established ones, knee joints were fixed in 4% paraformaldehyde overnight and then decalcified in 10% EDTA. Specimens were embedded in paraffin and sagittally sectioned at 5 μm thickness, followed by staining with 0.1% Safranin O to assess glycosaminoglycan (GAG) content and counterstaining with Gill’s hematoxylin and 0.02% Fast Green.

In addition, we performed limited optical assessments of an alternative OA model, i.e., a spontaneous chemically induced model using monosodium iodoacetate (MIA) treatment^29,88^, which has been well-established for recapitulating joint destruction and pain^89–92^. Compared with the DMM model, the MIA model induced histologically identifiable joint destruction at an earlier time, usually within 10 days. Upon MIA injection, there was a surge of inflammation, which subsequently subsided, as in the case of injury-induced OA^89–92^. To establish the MIA model, MIA (5 µg in 5 µL PBS) was directly injected into the knee joint of male C57B/L6 mice (18 weeks old)^29^. PBS injection served as a control. Injections took place daily for 7 days, when the knee joint was harvested, paraffin embedded, and sectioned for collagen organization analysis^93^.

We also collected human articular cartilage samples from the tibial plateaus of patients undergoing total knee replacement surgery for OA at Tufts Medical Center. The age, sex, and the Mankin scores of these donors were 63 (female, score: 7), 65 (female, score: 9), and 85 (male, score: 8), respectively. Unaffected regions (Mankin scores below 3) served as normal controls. Human specimens were processed in the same manner as for mouse ones, but were cryosectioned laterally at 40 µm thickness along the plane of the articular surface. Since the specimens were obtained from discarded joint pieces from knee replacement surgery, we were not able to obtain intact cartilage pieces as in early OA with transitional and radial zones consistently. Thus, samples were sectioned in parallel to the articular surface. Due to the more advanced age of the human subjects, the surface zone tended to be thinner and some cells imaged might be situated in the transitional zone. Human specimen acquisition protocols were reviewed by Tufts Institutional Review Board (IRB) and classified as exempt (IRB exempt number: STUDY00003258) because the samples were deidentified. All relevant ethical regulations were followed.

To validate the affected areas of human cartilage specimens, we collected samples from human cartilage donors from National Disease Research Interchange (NDRI) and performed Safranin O/Fast Green staining. OA affected and unaffected regions were first determined visually based on the smoothness of cartilage surface, as OA regions typically had fibrillated surface. Then neighboring areas were sectioned and subject to histological analysis with safranin O to confirm cartilage integrity and matrix levels, using the same staining protocols as the ones for mouse cartilage samples as mentioned above.

The impact of sucrose equilibration and cryosectioning of cartilage tissues on extracellular matrix was evaluated by comparing the collagen-associated fluorescence and SHG readouts from frozen and cryosectioned control or trypsin-treated porcine cartilage tissues. Porcine articular cartilage samples were obtained frozen from the local market. They were sliced to be 1 cm long and 0.5 cm wide and maintained at −20 °C prior to usage. To mimic cartilage degradation, porcine cartilage pieces were treated with trypsin-EDTA at 0.25% (Gibco, ThermoFisher) overnight, and then washed with medium to stop the reaction and stored in PBS until use. The porcine cartilage tissues were then processed in a similar way to the human cartilage tissues, i.e., equilibrated in a 30% sucrose solution for 3 days at 4 °C. Subsequently, the tissues were embedded in an OCT compound and cryosectioned laterally at 40 μm thickness. In addition, the impact of sucrose equilibration and cryosectioning of cartilage tissues on metabolic metrics was evaluated relying on comparisons between frozen and cryosectioned rodent epithelial tissue layers. The rodent epithelial tissues were processed in a similar way to mouse cartilage tissues. Specifically, they were excised immediately following euthanasia, snap frozen in liquid nitrogen, and kept at a −20 °C freezer until further use. For these experiments, they were removed from the freezer, imaged, and then equilibrated in a 30% sucrose solution for 3 days at 4 °C. Subsequently, they were embedded in an OCT compound and cryosectioned sagittally at 40 μm thickness.

Before multi-photon imaging, knee-joint slices were re-hydrated for 15 min in PBS and affixed on a glass slide using small drops of 50% glycerol solution and sealed with a coverslip secured in place with clear nail polish. During the optical imaging process, we focused on the cartilage of the tibia.

### Data acquisition

TPEF and SHG images were obtained using a Leica TCS SP8 confocal microscope equipped with a tunable (680–1300 nm) fs laser (InSight Deep See; Spectra Physics; Mountain View, CA). Images were acquired using a water-immersion 25× objective (NA 0.95; 2.4 mm working distance), with simultaneous collection by two non-descanned hybrid detectors (HyDs) using a filter cube containing filters from Chroma (Bellows Falls, VT), including a 700 nm short pass filter (ET700SP-2P) and a 495 nm dichroic mirror (495DCXR). To isolate NAD(P)H fluorescence, a 460 ± 20 nm emission filter (ET460/40M-2P), was placed before one of the non-descanned detectors. NAD(P)H fluorescence images were acquired using 755 nm excitation. Although NAD(P)H had a higher two-photon excitation action cross-section at shorter wavelengths^94^, this was the excitation-emission setting (755 nm ex./460 nm em.) that was used in our previous studies that validated optical redox ratio estimates to corresponding mass spectrometry ones^13,14^. At the time, this was the lowest wavelength provided by commercial Ti:sapphire lasers yielding sufficient illumination powers for cellular NAD(P)H signal detection. With current laser systems, lower excitation wavelengths are accessible and could enhance NAD(P)H signals and possibly our ability to detect optical redox ratio differences. FAD fluorescence was excited at 860 nm and isolated using a 525 ± 25 nm emission filter (ET525/50M-2P) placed in front of the other non-descanned detector. The two-photon action cross section of NAD(P)H decreased by several orders of magnitude between 755 nm and 860 nm excitation, enabling an efficient isolation of FAD at longer wavelengths. Endogenous fluorescence from collagen cross-links was collected by the 525 nm channel using 755 nm excitation, as in our previous study^30^. In addition to TPEF intensity, we also recorded the fluorescence lifetime (FLIM) of NAD(P)H and collagen cross-links using a time correlated single photon counting (TCSPC) system (Pico Harp 300) and SymPho software. SHG images were acquired using the 460 nm channel at 920 nm excitation. Detector gain was kept the same for the two HyDs and throughout all the experiments. The image intensity was normalized by the square of the incident laser power, as measured at the sample plane prior to each experiment. Picrosirius red-stained images of mouse cartilage samples were viewed using polarized light on a Zeiss Axioskop2 microscope. Except rodent epithelial tissues, 3D images of all the other tissue samples were acquired at a resolution of 512 × 512 pixels/386 × 386 µm, recorded for 40 μm depth with a step size of 1 μm. Frozen rodent epithelial tissues were imaged with a resolution of 512 × 512 pixels/232 × 232 µm, and cryosectioned ones were imaged with a resolution of 512 × 512 pixels/93 × 93 µm. For mouse DMM sample imaging, 4 tissue sections per group were prepared from each mouse (*n* = 4 mice), and 16 fields were imaged per mouse (4 fields per tissue section), yielding 64 image fields for each group at each of the five distinct time points (5 time points × 4 mice/group × 4 tissue sections/mouse × 4 fields/tissue section = 320 fields for full experiment). For human specimen imaging, 4 tissue sections per group were prepared from each patient (*n* = 3 patients), and at least 20 fields were imaged per patient (at least 5 fields per tissue section), which led to no less than 60 image fields for each of the control and OA groups (3 patients × 4 tissue sections/patient × 5 fields/tissue section). For mouse MIA sample imaging, 3 different samples per group were used and SHG images were acquired from 6 ROIs of control and 9 ROIs of MIA-treated mouse joints. For both porcine cartilage and rodent epithelial tissues, 3 different samples per group were used for the study and images were acquired from 3 ROIs per sample. Regarding the rodent epithelial tissues, images within 0–10 μm depth were considered from superficial tissues and images within 30–40 μm were considered from deeper tissues.

### Image pre-processing and segmentation between cells and extracellular matrix

For mouse cartilage analysis, we first rotated each image such that the articular surface was horizontally aligned, as described previously^27^. We then divided the cartilage region into four different zones based on the collagen fiber alignment. Collagen fibers aligned parallel to the articular surface in the superficial zone, but perpendicular to the surface in the radial zone. In the transitional zone, fibers were sinusoidal. The separation between the radial and the calcified zone was determined by the presence of the endplate, which presented itself as a dark line in SHG images. All the optical biomarkers proposed in this study were calculated in the superficial, transitional, and radial zones to provide zonal characteristics within cartilage. Within the cartilage region, collagen fibers demonstrated relatively strong signals under SHG imaging, while cells exhibited weak to dark SHG signals. Therefore, by intensity thresholding within the cartilage region of SHG images, we were able to separate matrix from cells, and perform quantitative analysis of matrix organization and cross-linking and cellular metabolism within the corresponding regions. Moreover, since the nucleus typically showed dark or weak signals in the NAD(P)H images and lipofuscin was intensely fluorescent, we performed intensity thresholding to identify the cytoplasm-only region (without the nucleus) and eliminate signals from lipofuscin for metabolic function analysis. For human and porcine cartilage specimen analysis, we performed the same pre-processing procedures except that we did not need to rotate the image nor perform the zonal segmentation since images were acquired parallel to the surface. For rodent epithelial tissues, intensity thresholding was employed to identify the cytoplasm-only region for cell metabolic analysis.

### 3D directional variance assessments

We have previously described in detail the methods we developed to assess 3D orientation and 3D organization for fiber-like structures^27,95^. The 3D orientation algorithm was based on an extension of a previously established 2D weighted vector summation technique^96^, and yielded values of the azimuthal angle $\theta$ and the polar angle $\varphi$ for each voxel using a user-specified window size (Supplementary Fig. 1a, b). The 3D directional variance (Supplementary Fig. 1c, d) was generated based on the 3D fiber orientations^27^, and its value varied between 0 and 1, with 0 indicating perfectly parallel fiber alignment, while 1 corresponding to completely random organization. The custom code used for assessing 3D orientation and organization of fiber-like structures was developed in MATLAB and available for download at: https://engineering.tufts.edu/bme/georgakoudi/publications.

To calculate the 3D orientation and 3D directional variance of a certain voxel of the collagen fiber stack image, as represented by the SHG signal, we first created an $n\times n\times m$ voxel window surrounding this voxel, and the SHG intensity information within this window was used to determine its orientation and organization^95^. Here the numbers of $n$ and $m$ for the window size were chosen based on the axial-to-lateral sampling ratio, so as to guarantee an approximately cubic sized window. According to our previous studies, a window of two to three times the fiber diameter led to optimized accuracy orientation results^95^. In this study, to assess the localized 3D directional variance, we used 5.3 × 5.3 × 5 µm as the window size for both orientation and 3D directional variance calculations, since the diameter of collagen fibers was typically 1–2 µm. While distortions introduced by the elongated point spread function of our images in the direction of light propagation may impact the absolute accuracy of the 3D variance assessments, such effects impact imaging and analysis of all specimens and should not affect the nature of the reported differences between groups of samples.

### Cross-link content analysis

TPEF intensity signal acquired at 755 nm excitation and 525 ± 25 nm emission within the SHG positive pixels was attributed to collagen cross-links. The TPEF signal was further normalized by SHG signal as a more robust biomarker of cross-link density. The cross-link density maps were color-coded in MATLAB for visualization purposes. The mean cross-link density level was acquired by averaging the pixel-wise values within the collagen-only area.

### Phasor-based fluorescence lifetime analysis

We acquired fluorescence lifetime images at 755 nm excitation, using the 460 nm emission channel corresponding to cellular NAD(P)H, and the 525 nm emission corresponding to matrix cross-links. Then, real and imaginary parts of the Fourier transform of the decay curve at each pixel were used to determine the two coordinates of a phasor, as previously defined^97^. For such a transformation the fluorescence lifetime profile characterized by a mono-exponential decay was mapped onto a point that fell on the universal semicircle of the phasor plot, while more complicated decay curves were represented by points within the semicircle (Supplementary Fig. 6a, c). Fluorescence lifetime increased in the counter-clockwise direction along the reference arc. If a fluorescence decay curve was well-described by a bi-exponential function, its phasor fell on a line within the semicircle, and the two points where the line intersected the semicircle represented the short- (right intersection) and long-lifetime (left intersection) components (Supplementary Fig. 6a, c). The relative position of the point along that line can provide an estimate of the fractional contributions of the short- and long-lifetime components^15,19^. Throughout this study, we used the long-lifetime intensity fraction (LLIF), estimated based on the location of the centroid of corresponding phasor plots, as a quantitative measure to resolve both cross-link (Supplementary Fig. 6b) and NAD(P)H (Supplementary Fig. 6d) fluorescence lifetime information. The LLIF can be quantified for each pixel, yielding color-coded LLIF image maps. The mean LLIF of cross-links or NAD(P)H was acquired by averaging the values within only the ECM or the cellular cytoplasm area, respectively.

### Redox ratio calculation

The optical redox ratio was calculated based on the TPEF image intensity ratio of FAD/(NAD(P)H + FAD) (Supplementary Fig. 7). The redox ratio maps were color-coded in MATLAB and multiplied by merged grayscale intensity images of NAD(P)H and FAD for visualization purposes. The mean redox ratio was acquired by averaging the values within the cellular cytoplasm area.

### Mitochondrial clustering calculation

Assessment of mitochondrial clustering was performed using our previously established, automated Fourier-based approach, which relied on the fact that the quantum efficiency of NAD(P)H fluorescence was enhanced by ten-fold when NAD(P)H was bound within the mitochondria^17,98^. Briefly, the NAD(P)H image intensity patterns (Supplementary Fig. 8a) within the cytoplasm-only regions (Supplementary Fig. 8b) of each field were selected and used for the subsequent clone-stamping (i.e., randomly positioning these selected intensity patterns in the portion of the image originally occupied by nuclei and inter-cellular areas) to create a new one without distinct cell and nuclear borders and only cell mitochondria patterns (Supplementary Fig. 8c)^98,99^. Then, we acquired the power spectral density (PSD) of the 2D Fourier transform of the clone-stamped image, and fitted an inverse power law expression to the portion of the spectrum between 0.1 μm^−1^ and a high-frequency cut-off eliminating 2% of the signal (this signal was attributed to noise). The value of the power law exponent, β, was an indicator of the mitochondrial clustering levels (Supplementary Fig. 8d). Each random clone-stamping operation might generate a slightly different image, thus yielding a slightly different β value. To address this issue, we repeated the clone-stamping operation 20 times and used the average β value as a representative of the mitochondrial clustering for each image.

### Metabolic function 3D scatterplot preparation

In order to gain insights into cellular metabolic function changes during the progression of OA at early stages, we mapped the relative changes of the three metabolic metrics, i.e., optical redox ratio, NAD(P)H LLIF, and mitochondrial clustering, into the corresponding 3D space with our previously validated database of changes in these metrics attributed to seven important metabolic perturbations, including glycolysis and glutaminolysis/oxidative phosphorylation, extrinsic and intrinsic mitochondrial uncoupling, and fatty acid oxidation (saturated and unsaturated substrates) and synthesis^19^. To achieve this, we acquired the mean values of these three metrics for each image, calculated the changes of each metric caused by DMM relative to the corresponding sham mouse surgery specimens, or OA relative to “normal” human specimens, and mapped them into the 3D space, with each of the three coordinates corresponding to one optical metric. These 3D scatterplots provided an intuitive impression of the possible dynamic metabolic changes attributed to OA development, and enabled visualization of field-to-field metabolic heterogeneity.

### viSNE for visualization of classification

The viSNE dimensionality reduction tool was used to visualize the separation between DMM (OA) and sham (normal) specimens when considering all six optical biomarkers: 3D directional variance, cross-link density, cross-link LLIF, redox ratio, NAD(P)H LLIF and mitochondrial clustering. The viSNE method was initially developed for dealing with cellular heterogeneity issues, but we extended it to a more general application (e.g., field-based data in this case). These field-based data were analyzed in Cytobank (www.cytobank.org) to create a viSNE map^100,101^. viSNE performed t-distributed stochastic neighbor embedding (t-SNE) to minimize the differences between high-dimensional space and low-dimensional space, and produced a 2D plot in arbitrary units. Briefly, a pairwise distance matrix was calculated in high-dimensional space, which was transformed to a low-dimensional similarity matrix. The points were randomly mapped in low-dimensional space and iteratively rearranged to minimize the divergence between high-dimensional and low-dimensional similarity matrices^101^. To demonstrate the ability to identify specific time-dependent changes in articular cartilage following OA-induction in the DMM mouse model using a combination of all the six optical biomarkers, we generated a global viSNE map including all the time points from the mouse DMM data, as well as separate maps for mouse and human data. Moreover, the distributions of each optical biomarker across all the data points on the viSNE map were included to indicate how well a certain biomarker performed in distinguishing DMM (OA) from sham (normal).

### Statistics and reproducibility

An ANOVA with post hoc Tukey HSD test was performed to assess significant differences for mouse and porcine articular cartilage tissues and rodent epithelial tissues using JMP 15 (SAS Institute). Comparisons from human specimens were done using a two-tailed t-test. Results were considered significant at *p* < 0.05. The experimental data were expressed as mean ± SD for four mice (*n* = 4) from each group at each time point and three patients (*n* = 3). To evaluate the separation model using a combination of optical biomarkers, canonical linear discriminant analysis was performed. Discrimination accuracies were calculated with the linear discriminant functions determined and applied using the entire data set (corresponding to OCA value) and a leave-one-out cross-validation scheme (corresponding to CVCA value) via SPSS. To ensure lack of offending variables and independence of these six variables, multivariate analysis was implemented to evaluate multicollinearity between these variables for mouse data from distinct time points and human data using the Pearson product-moment correlation coefficient r. Pearson correlation coefficients between variables were reported on the basis of the null hypothesis that *r* = 0.

### Reporting summary

Further information on research design is available in the Nature Portfolio Reporting Summary linked to this article.

## Supplementary information

Supplementary Information

Description of Additional Supplementary Files

Supplementary Data 1

Supplementary Video 1

Supplementary Video 2

Reporting Summary

## Data Availability

The source data that make up all graphs in the paper are shown in Supplementary Data 1. Any further data that support the findings of this study are available from the corresponding author upon request.

## Code availability

The custom code used for assessing 3D orientation and organization of fiber-like structures was developed in MATLAB and available for download at: https://engineering.tufts.edu/bme/georgakoudi/publications, as well as the Zenodo platform^102^.

## References

[1] Kamekura S (2005). Osteoarthritis development in novel experimental mouse models induced by knee joint instability. Osteoarthr. Cartil..

[2] Breedveld FC (2004). Osteoarthritis—the impact of a serious disease. Rheumatology.

[3] Felson DT (2000). Osteoarthritis: new insights. Part 1: the disease and its risk factors. Ann. Intern Med..

[4] Fukui N (2008). Regional differences in chondrocyte metabolism in osteoarthritis: a detailed analysis by laser capture microdissection. Arthritis Rheum..

[5] Saarakkala S (2010). Depth-wise progression of osteoarthritis in human articular cartilage: investigation of composition, structure and biomechanics. Osteoarthr. Cartil..

[6] Manning HB (2013). Detection of cartilage matrix degradation by autofluorescence lifetime. Matrix Biol..

[7] Zhuo Q, Yang W, Chen J, Wang Y (2012). Metabolic syndrome meets osteoarthritis. Nat. Rev. Rheumatol..

[8] Lai WF, Chang CH, Tang Y, Bronson R, Tung CH (2004). Early diagnosis of osteoarthritis using cathepsin B sensitive near-infrared fluorescent probes. Osteoarthr. Cartil..

[9] Denk W, Strickler JH, Webb WW (1990). Two-photon laser scanning fluorescence microscopy. Science.

[10] Georgakoudi I, Quinn KP (2012). Optical imaging using endogenous contrast to assess metabolic state. Annu. Rev. Biomed. Eng..

[11] Skala MC (2007). In vivo multiphoton microscopy of NADH and FAD redox states, fluorescence lifetimes, and cellular morphology in precancerous epithelia. Proc. Natl Acad. Sci. USA.

[12] Liu ZY (2021). Nicotinamide effects on the metabolism of human fibroblasts and keratinocytes assessed by quantitative, label-free fluorescence imaging. Biomed. Opt. Express.

[13] Varone A (2014). Endogenous two-photon fluorescence imaging elucidates metabolic changes related to enhanced glycolysis and glutamine consumption in precancerous epithelial tissues. Cancer Res..

[14] Quinn KP (2013). Quantitative metabolic imaging using endogenous fluorescence to detect stem cell differentiation. Sci. Rep..

[15] Alonzo CA (2016). Two-photon excited fluorescence of intrinsic fluorophores enables label-free assessment of adipose tissue function. Sci. Rep..

[16] Stuntz E (2017). Endogenous two-photon excited fluorescence imaging characterizes neuron and astrocyte metabolic responses to manganese toxicity. Sci. Rep..

[17] Levitt JM (2007). Diagnostic cellular organization features extracted from autofluorescence images. Opt. Lett..

[18] Pouli D (2016). Imaging mitochondrial dynamics in human skin reveals depth-dependent hypoxia and malignant potential for diagnosis. Sci. Transl. Med..

[19] Liu, Z. et al. Mapping metabolic changes by noninvasive, multiparametric, high-resolution imaging using endogenous contrast. *Sci. Adv.* **4**, eaap9302 (2018). 10.1126/sciadv.aap9302 PMC5846284 29536043

[20] Poole AR (2002). Type II collagen degradation and its regulation in articular cartilage in osteoarthritis. Ann. Rheum. Dis..

[21] Chaudhary R (2015). Articular cartilage zonal differentiation via 3D Second-Harmonic Generation imaging microscopy. Connect Tissue Res..

[22] Zhang Y (2012). A compact fiber-optic SHG scanning endomicroscope and its application to visualize cervical remodeling during pregnancy. Proc. Natl Acad. Sci. USA.

[23] Campagnola PJ, Loew LM (2003). Second-harmonic imaging microscopy for visualizing biomolecular arrays in cells, tissues and organisms. Nat. Biotechnol..

[24] Kim JH (2015). Matrix cross-linking-mediated mechanotransduction promotes posttraumatic osteoarthritis. Proc. Natl Acad. Sci. USA.

[25] Marturano JE, Xylas JF, Sridharan GV, Georgakoudi I, Kuo CK (2014). Lysyl oxidase-mediated collagen crosslinks may be assessed as markers of functional properties of tendon tissue formation. Acta Biomater..

[26] Hughes LC, Archer CW, AP Gwynn I (2005). The ultrastructure of mouse articular cartilage: collagen orientation and implications for tissue functionality. A polarised light and scanning electron microscope study and review. Eur. Cell Mater..

[27] Liu Z (2017). Automated quantification of three-dimensional organization of fiber-like structures in biological tissues. Biomaterials.

[28] Nieminen MT (2001). T2 relaxation reveals spatial collagen architecture in articular cartilage: a comparative quantitative MRI and polarized light microscopic study. Magn. Reson Med..

[29] Pitcher T, Sousa-Valente J, Malcangio M (2016). The monoiodoacetate model of osteoarthritis pain in the mouse. J. Vis. Exp..

[30] Huang M (2018). Lysyl oxidase enzymes mediate TGF-beta1-induced fibrotic phenotypes in human skin-like tissues. Lab Invest..

[31] Schweitzer D (2007). Towards metabolic mapping of the human retina. Microsc. Res. Tech..

[32] Alves CJ (2020). Nociceptive mechanisms driving pain in a post-traumatic osteoarthritis mouse model. Sci. Rep..

[33] Hui Mingalone CK (2018). Bioluminescence and second harmonic generation imaging reveal dynamic changes in the inflammatory and collagen landscape in early osteoarthritis. Lab Invest..

[34] McNulty MA (2011). A comprehensive histological assessment of osteoarthritis lesions in mice. Cartilage.

[35] Zipfel WR (2003). Live tissue intrinsic emission microscopy using multiphoton-excited native fluorescence and second harmonic generation. Proc. Natl Acad. Sci. USA.

[36] Kielty CM, Sherratt MJ, Shuttleworth CA (2002). Elastic fibres. J. Cell Sci..

[37] Mansfield JC, Mandalia V, Toms A, Winlove CP, Brasselet S (2019). Collagen reorganization in cartilage under strain probed by polarization sensitive second harmonic generation microscopy. J. R. Soc. Interface.

[38] Chu CR, Williams AA, Coyle CH, Bowers ME (2012). Early diagnosis to enable early treatment of pre-osteoarthritis. Arthritis Res. Ther..

[39] Desrochers J, Amrein MW, Matyas JR (2012). Viscoelasticity of the articular cartilage surface in early osteoarthritis. Osteoarthr. Cartil..

[40] Casula V (2017). Elevated adiabatic T1rho and T2rho in articular cartilage are associated with cartilage and bone lesions in early osteoarthritis: a preliminary study. J. Magn. Reson Imaging.

[41] Bi X (2007). Fourier transform infrared imaging and MR microscopy studies detect compositional and structural changes in cartilage in a rabbit model of osteoarthritis. Anal. Bioanal. Chem..

[42] Hyttinen MM (2001). Age matters: collagen birefringence of superficial articular cartilage is increased in young guinea-pigs but decreased in older animals after identical physiological type of joint loading. Osteoarthr. Cartil..

[43] Koff MF (2013). Correlation of meniscal T2* with multiphoton microscopy, and change of articular cartilage T2 in an ovine model of meniscal repair. Osteoarthr. Cartil..

[44] Drexler W (2001). Correlation of collagen organization with polarization sensitive imaging of in vitro cartilage: implications for osteoarthritis. J. Rheumatol..

[45] Cillero-Pastor B, Eijkel GB, Kiss A, Blanco FJ, Heeren RM (2013). Matrix-assisted laser desorption ionization-imaging mass spectrometry: a new methodology to study human osteoarthritic cartilage. Arthritis Rheum..

[46] Venkatesan N (2012). Xylosyltransferase-I regulates glycosaminoglycan synthesis during the pathogenic process of human osteoarthritis. PLoS ONE.

[47] Bi, Y., Patra, P. & Faezipour, M. Structure of c ollagen-glycosaminoglycan matrix and the influence to its integrity and stability. in *2014 36th Annual International Conference of the IEEE Engineering in Medicine and Biology Society* 3949–3952 (2014). 10.1109/EMBC.2014.6944488 25570856

[48] Liu Z (2018). 3D organizational mapping of collagen fibers elucidates matrix remodeling in a hormone-sensitive 3D breast tissue model. Biomaterials.

[49] Eyre DR, Wu JJ (2005). Collagen cross-links. Top. Curr. Chem..

[50] Huang M (2020). Systemic sclerosis dermal fibroblasts induce cutaneous fibrosis through lysyl oxidase-like 4: new evidence from three-dimensional skin-like tissues. Arthritis Rheumatol..

[51] Doyran B (2017). Nanoindentation modulus of murine cartilage: a sensitive indicator of the initiation and progression of post-traumatic osteoarthritis. Osteoarthr. Cartil..

[52] Rambold AS, Kostelecky B, Elia N, Lippincott-Schwartz J (2011). Tubular network formation protects mitochondria from autophagosomal degradation during nutrient starvation. Proc. Natl Acad. Sci. USA.

[53] Amaravadi R, Kimmelman AC, White E (2016). Recent insights into the function of autophagy in cancer. Genes Dev..

[54] Lane RS (2015). Mitochondrial respiration and redox coupling in articular chondrocytes. Arthritis Res. Ther..

[55] Zhang L, Hu J, Athanasiou KA (2009). The role of tissue engineering in articular cartilage repair and regeneration. Crit. Rev. Biomed. Eng..

[56] Mobasheri A (2017). The role of metabolism in the pathogenesis of osteoarthritis. Nat. Rev. Rheumatol..

[57] Ziskoven C (2010). Oxidative stress in secondary osteoarthritis: from cartilage destruction to clinical presentation?. Orthop. Rev..

[58] Simon HU, Haj-Yehia A, Levi-Schaffer F (2000). Role of reactive oxygen species (ROS) in apoptosis induction. Apoptosis.

[59] Wang HW (2008). Differentiation of apoptosis from necrosis by dynamic changes of reduced nicotinamide adenine dinucleotide fluorescence lifetime in live cells. J. Biomed. Opt..

[60] Wang Y, Nartiss Y, Steipe B, McQuibban GA, Kim PK (2012). ROS-induced mitochondrial depolarization initiates PARK2/PARKIN-dependent mitochondrial degradation by autophagy. Autophagy.

[61] Elzinga EH (2007). 2-Deoxy-2-[F-18]fluoro-D-glucose joint uptake on positron emission tomography images: rheumatoid arthritis versus osteoarthritis. Mol. Imaging Biol..

[62] Bhattacharyya T (2003). The clinical importance of meniscal tears demonstrated by magnetic resonance imaging in osteoarthritis of the knee. J. Bone Jt. Surg. Am..

[63] Pifferi A (2004). Optical biopsy of bone tissue: a step toward the diagnosis of bone pathologies. J. Biomed. Opt..

[64] Hielscher AH (2004). Sagittal laser optical tomography for imaging of rheumatoid finger joints. Phys. Med. Biol..

[65] Yuan Z, Zhang Q, Sobel E, Jiang H (2007). Three-dimensional diffuse optical tomography of osteoarthritis: initial results in the finger joints. J. Biomed. Opt..

[66] Yuan Z, Jiang H (2009). Quantitative photoacoustic tomography. Philos. Trans. A Math. Phys. Eng. Sci..

[67] Laufer J, Delpy D, Elwell C, Beard P (2007). Quantitative spatially resolved measurement of tissue chromophore concentrations using photoacoustic spectroscopy: application to the measurement of blood oxygenation and haemoglobin concentration. Phys. Med. Biol..

[68] Sun Y, Sobel ES, Jiang H (2011). First assessment of three-dimensional quantitative photoacoustic tomography for in vivo detection of osteoarthritis in the finger joints. Med. Phys..

[69] Calamia V (2011). Metabolic labeling of chondrocytes for the quantitative analysis of the interleukin-1-beta-mediated modulation of their intracellular and extracellular proteomes. J. Proteome Res..

[70] Buchberger AR, DeLaney K, Johnson J, Li LJ (2018). Mass spectrometry imaging: a review of emerging advancements and future insights. Anal. Chem..

[71] Gamble LJ, Anderton CR (2016). Secondary ion mass spectrometry imaging of tissues, cells, and microbial systems. Micros Today.

[72] Aichler M, Walch A (2015). MALDI Imaging mass spectrometry: current frontiers and perspectives in pathology research and practice. Lab Invest.

[73] Maldonado, M. & Nam, J. The role of changes in extracellular matrix of cartilage in the presence of inflammation on the pathology of osteoarthritis. *Biomed Res. Int.* **2013**, 284873 (2013). 10.1155/2013/284873 PMC3771246 24069595

[74] Soto-Heredero G, Gomez de Las Heras MM, Gabande-Rodriguez E, Oller J, Mittelbrunn M (2020). Glycolysis—a key player in the inflammatory response. FEBS J..

[75] Palsson-McDermott EM, O’Neill LAJ (2020). Targeting immunometabolism as an anti-inflammatory strategy. Cell Res..

[76] Wilkins RJ, Hall AC (1995). Control of matrix synthesis in isolated bovine chondrocytes by extracellular and intracellular pH. J. Cell Physiol..

[77] Zheng L, Zhang Z, Sheng P, Mobasheri A (2021). The role of metabolism in chondrocyte dysfunction and the progression of osteoarthritis. Ageing Res. Rev..

[78] DeGroot J (2004). Advanced glycation endproducts in the development of osteoarthritis. Ann. Rheum. Dis..

[79] Saudek DM, Kay J (2003). Advanced glycation endproducts and osteoarthritis. Curr. Rheumatol. Rep..

[80] Yang Q (2015). Advanced glycation end products-induced chondrocyte apoptosis through mitochondrial dysfunction in cultured rabbit chondrocyte. Fundam. Clin. Pharm..

[81] Wu S, Zhou F, Zhang Z, Xing D (2011). Mitochondrial oxidative stress causes mitochondrial fragmentation via differential modulation of mitochondrial fission-fusion proteins. FEBS J..

[82] Lee YR (2020). Mass spectrometry imaging as a potential tool to investigate human osteoarthritis at the tissue level. Int J. Mol. Sci..

[83] Antonini A (2020). Extended field-of-view ultrathin microendoscopes for high-resolution two-photon imaging with minimal invasiveness. Elife.

[84] Glasson SS, Blanchet TJ, Morris EA (2007). The surgical destabilization of the medial meniscus (DMM) model of osteoarthritis in the 129/SvEv mouse. Osteoarthr. Cartil..

[85] Gibson AL (2017). Wnt7a inhibits IL-1beta induced catabolic gene expression and prevents articular cartilage damage in experimental osteoarthritis. Sci. Rep..

[86] Milazzo JP (2010). Rapid screening of cryopreservation protocols for murine prepubertal testicular tissue by histology and PCNA immunostaining. J. Androl..

[87] Rittie L (2017). Method for picrosirius red-polarization detection of collagen fibers in tissue sections. Methods Mol. Biol..

[88] Moilanen LJ (2015). Monosodium iodoacetate-induced inflammation and joint pain are reduced in TRPA1 deficient mice–potential role of TRPA1 in osteoarthritis. Osteoarthr. Cartil..

[89] Guingamp C (1997). Mono-iodoacetate-induced experimental osteoarthritis: a dose-response study of loss of mobility, morphology, and biochemistry. Arthritis Rheum..

[90] Guzman RE, Evans MG, Bove S, Morenko B, Kilgore K (2003). Mono-iodoacetate-induced histologic changes in subchondral bone and articular cartilage of rat femorotibial joints: an animal model of osteoarthritis. Toxicol. Pathol..

[91] Haslauer CM, Proffen BL, Johnson VM, Hill A, Murray MM (2014). Gene expression of catabolic inflammatory cytokines peak before anabolic inflammatory cytokines after ACL injury in a preclinical model. J. Inflamm..

[92] Johnson K (2004). Mediation of spontaneous knee osteoarthritis by progressive chondrocyte ATP depletion in Hartley guinea pigs. Arthritis Rheum..

[93] Uchimura T (2019). Erythromycin acts through the ghrelin receptor to attenuate inflammatory responses in chondrocytes and maintain joint integrity. Biochem. Pharm..

[94] Huang S, Heikal AA, Webb WW (2002). Two-photon fluorescence spectroscopy and microscopy of NAD(P)H and flavoprotein. Biophys. J..

[95] Liu Z (2015). Rapid three-dimensional quantification of voxel-wise collagen fiber orientation. Biomed. Opt. Express.

[96] Quinn KP, Georgakoudi I (2013). Rapid quantification of pixel-wise fiber orientation data in micrographs. J. Biomed. Opt..

[97] Digman MA, Caiolfa VR, Zamai M, Gratton E (2008). The phasor approach to fluorescence lifetime imaging analysis. Biophys. J..

[98] Xylas J, Quinn KP, Hunter M, Georgakoudi I (2012). Improved Fourier-based characterization of intracellular fractal features. Opt. Express.

[99] Xylas J (2015). Noninvasive assessment of mitochondrial organization in three-dimensional tissues reveals changes associated with cancer development. Int. J. Cancer.

[100] Amir el AD (2013). viSNE enables visualization of high dimensional single-cell data and reveals phenotypic heterogeneity of leukemia. Nat. Biotechnol..

[101] Shah AT, Diggins KE, Walsh AJ, Irish JM, Skala MC (2015). In vivo autofluorescence imaging of tumor heterogeneity in response to treatment. Neoplasia.

[102] Liu, Z. et al. Code used for assessing 3D orientation and organization of fiber-like structures. *Zenodo*. 10.5281/zenodo.7669471 (2023).

