# Supplementary material for: Label-free, multi-parametric assessments of cell metabolism and matrix remodeling within human and early-stage murine osteoarthritic articular cartilage
Source: Commun Biol. 2023 Apr 13;6:405. doi: 10.1038/s42003-023-04738-w (PMC10102009; doi:10.1038/s42003-023-04738-w)
Supplement: Supplementary file 1 — Supplementary Information [file 42003_2023_4738_MOESM1_ESM.pdf]

Supplementary Materials for

# Label-free, multi-parametric assessments of cell metabolism and matrix remodeling within human and early-stage murine osteoarthritic articular cartilage

Zhiyi Liu,† Carrie K. Hui Mingalone,† Einstein Gnanatheepam, Judith M. Hollander, Yang Zhang, Jia Meng, Li Zeng, Irene Georgakoudi*

[†]These authors contributed equally

[*]Correspondence to: Irene.Georgakoudi@tufts.edu

This PDF file includes:

Supplementary Figures 1-20

Supplementary Table 1

**Supplementary Figures:**

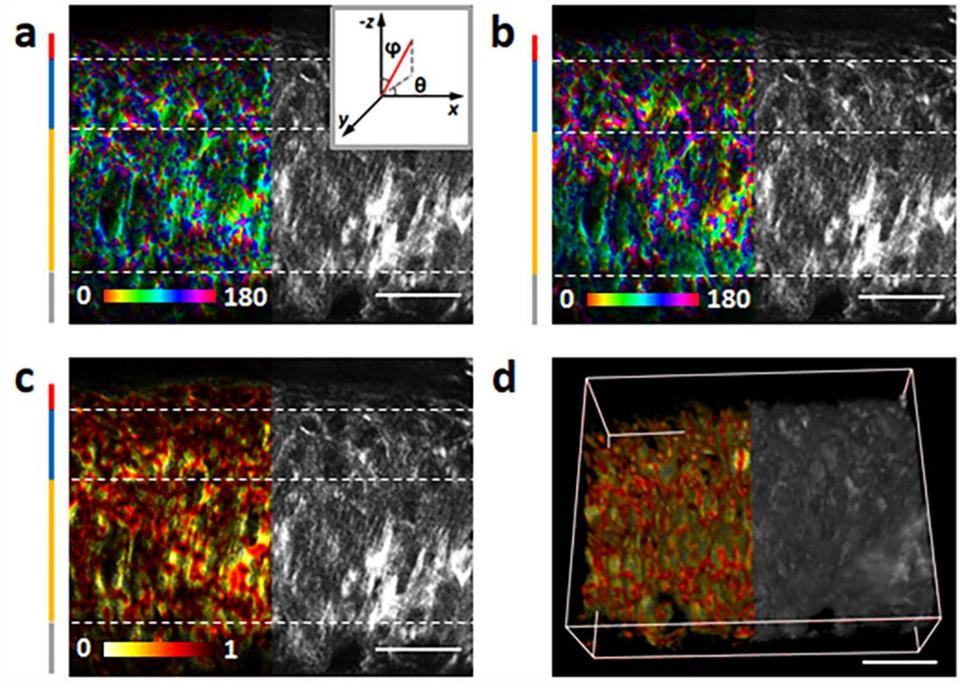


**Supplementary Fig. 1. Schematics demonstrating the calculation of 3D organization of collagen fibers. a**, The $\theta$ orientation map of a representative field is shown in the left half. The right half shows the raw SHG image of collagen fibers. To depict a direction in 3D space, an azimuthal angle $\theta$ and a polar angle $\varphi$ are defined, as indicated in the inset. **b**, The $\varphi$ orientation map of the same field. **c**, The corresponding 3D directional variance map showing the spatial organization of collagen fibers. **d**, The 3D directional variance map of the entire 3D stack. Scale bar: 30 µm.

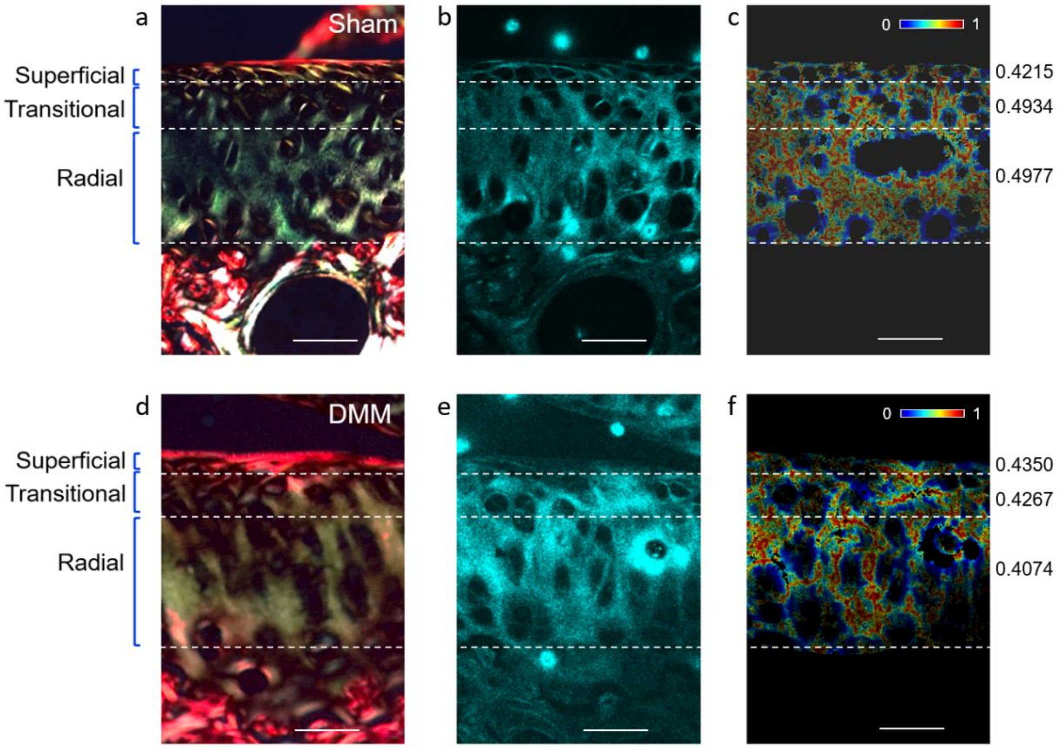


**Supplementary Fig. 2. Validation of zone segmentation by picrosirius red-polarization detection.** Representative **a**, picrosirius red stained image, **b**, SHG image and **c**, corresponding 3D directional variance map of the sham mouse sample at 10 weeks post-surgery. Representative **d**, picrosirius red stained image, **e**, SHG image and **f**, corresponding 3D directional variance map of the DMM mouse sample at 10 weeks post-surgery. 3D directional variance values for each zone are indicated on the right of the variance maps. Scale bar: 50 μm.

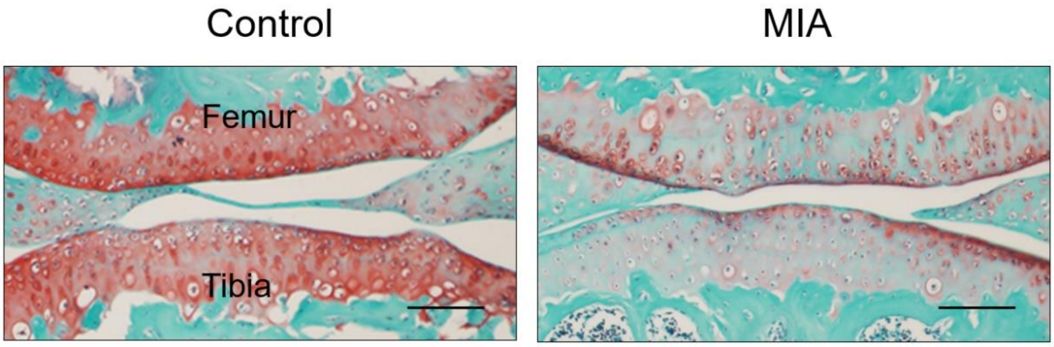


**Supplementary Fig. 3. Safranin O/Fast Green staining of PBS control and MIA treated mouse knee sections.** Scale bar: 400 μm.

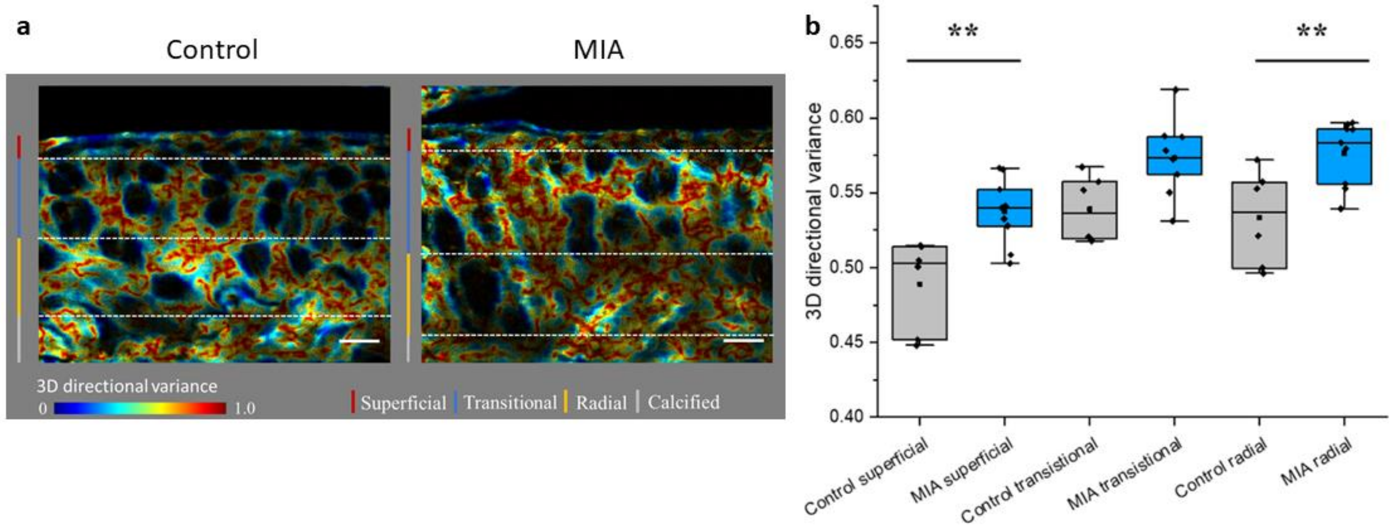


**Supplementary Fig. 4. Representative maps and analysis results following MIA treatment. a**, Representative directional variance maps of cartilage from PBS control and MIA treated mouse joint tissues. **b**, Field-based boxplots of 3D directional variance of control and MIA treated mouse joint tissue samples at different zones. **, $p < 0.01$. Scale bar: 50 µm.

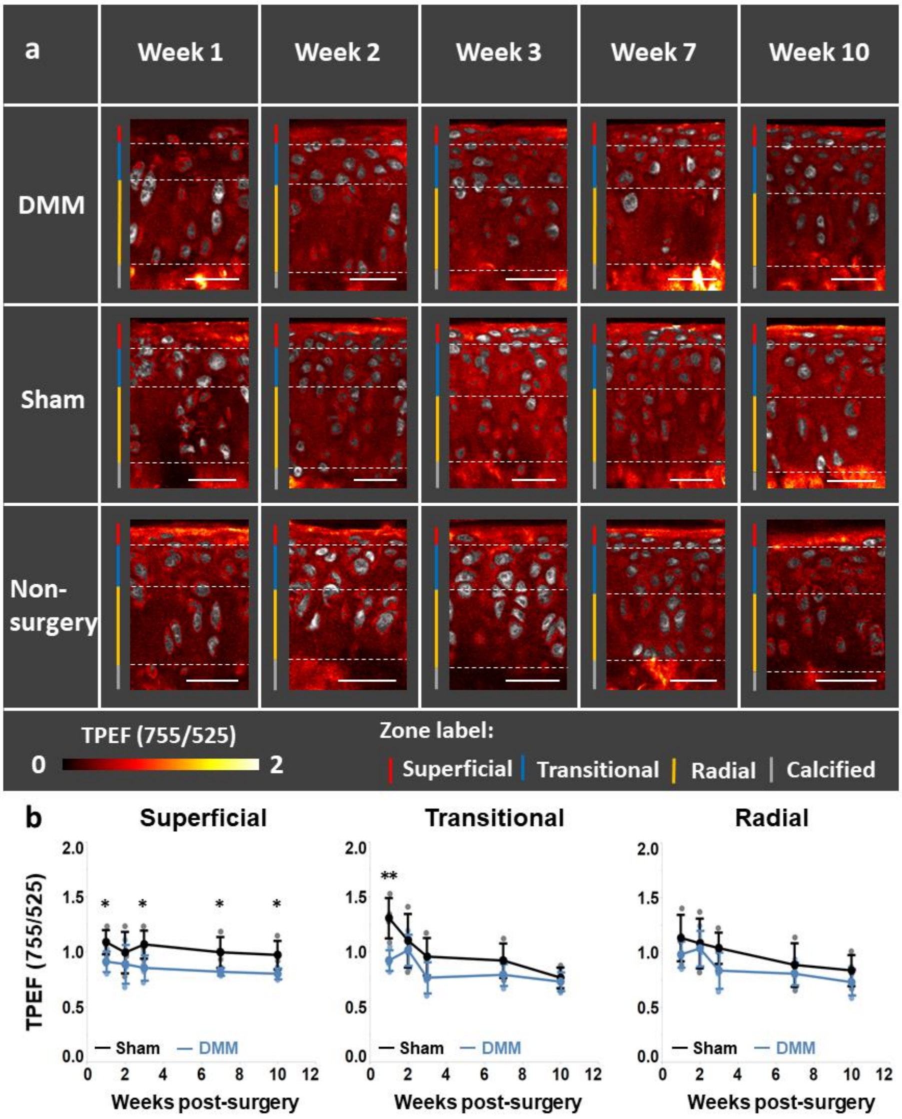


**Supplementary Fig. 5. Raw crosslink fluorescence as represented by TPEF signal decreases in DMM specimens compared with that of sham specimens. a**, Representative maps of raw crosslink fluorescence from DMM (top), sham (middle), and non-surgery control (bottom) at 1, 2, 3, 7 and 10 weeks post-treatment. **b**, The mean and standard deviation of raw crosslink fluorescence normalized to non-surgery controls at different time points and distinct zones. *, $p < 0.05$ and **, $p < 0.01$. Scale bar: 30 μm.

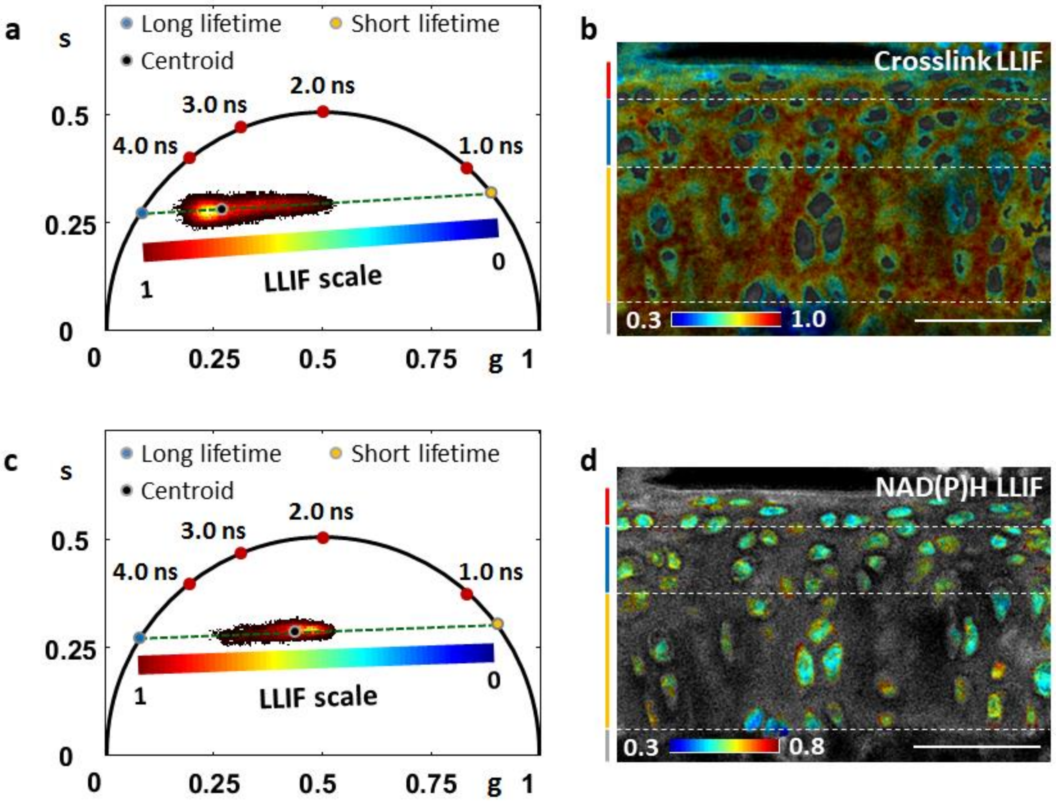


**Supplementary Fig. 6. Phasor plot and representative long lifetime intensity fraction (LLIF) maps. a**, A representative phasor plot for crosslinks. The *x* and *y* axis in the phasor plot represent the real (g) and imaginary (s) parts of the Fourier transform of the decay curve. **b**, Representative crosslink LLIF map. **c**, A representative phasor plot for NAD(P)H. **d**, Representative NAD(P)H LLIF map. Scale bar: 50 μm.

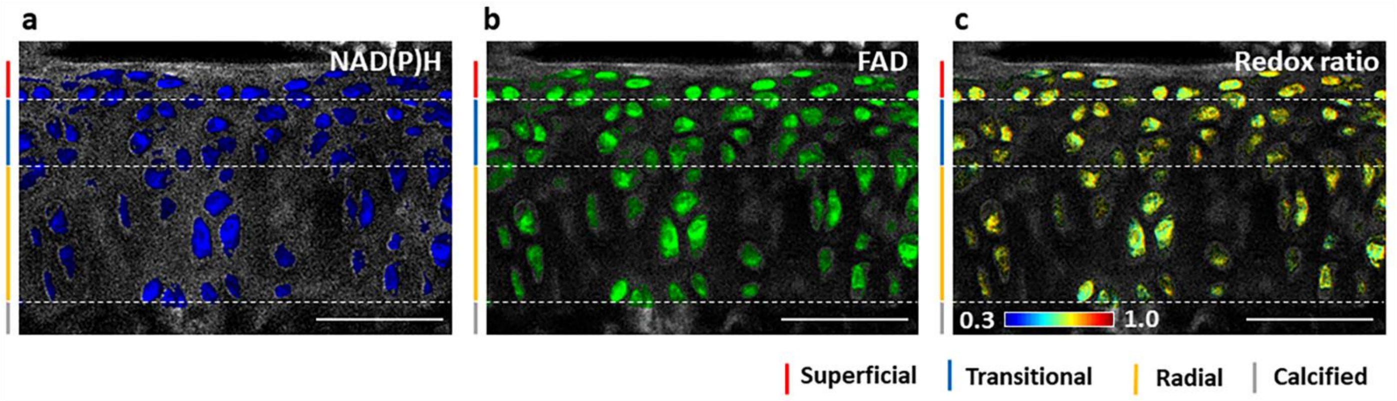


**Supplementary Fig. 7. Schematic showing the calculation of the optical redox ratio. a**, A representative blue-coded NAD(P)H image. We note that only the cell region is color-coded, and the collagen region is shown by gray hues for visualization purposes. **b**, The corresponding green-coded FAD image. **c**, The corresponding redox ratio map as defined by: FAD/ (NAD(P)H + FAD). Scale bar: 50 µm.

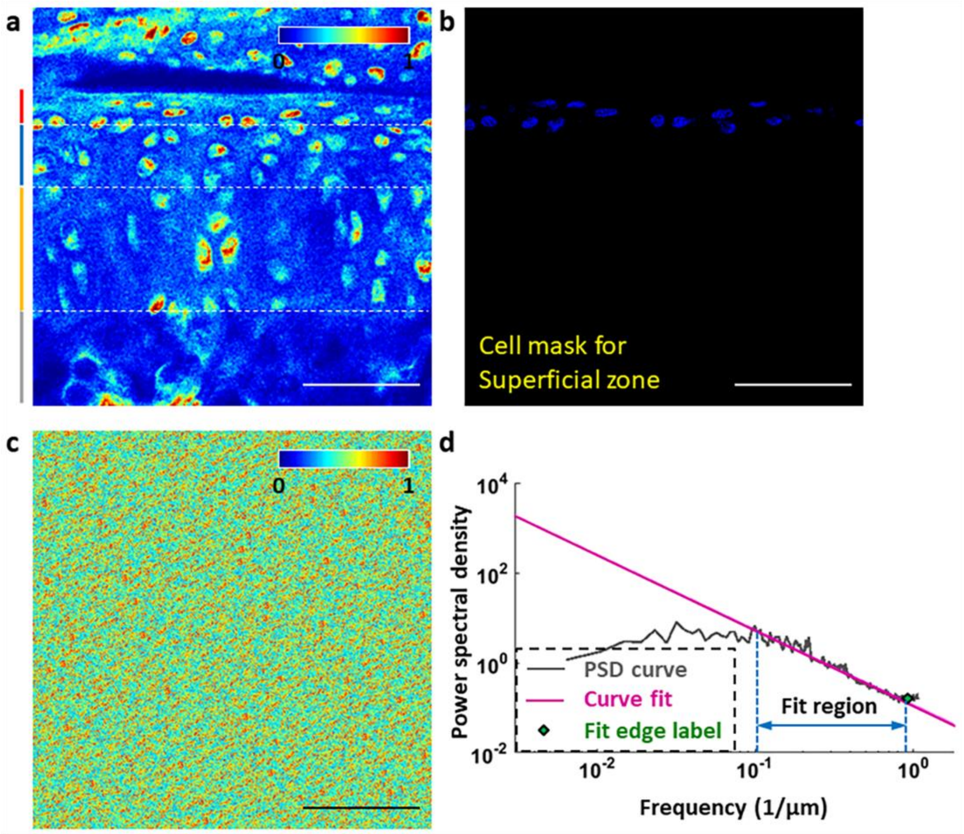


**Supplementary Fig. 8. Schematic showing the calculation of mitochondrial clustering. a**, A representative NAD(P)H TPEF intensity image. **b**, The corresponding mask (blue-colored) selecting cells in the superficial zone. **c**, The clone-stamped image of the cell patterns within the mask shown in **b**. **d**, Calculation of mitochondrial clustering based on the clone-stamped image. Upon Fourier transformation, the power spectral density (PSD) curve (gray) is acquired, and then the linear portion is fitted (magenta) to obtain the exponential power, β, which is an indicator of the mitochondrial clustering. Scale bar: 100 μm.

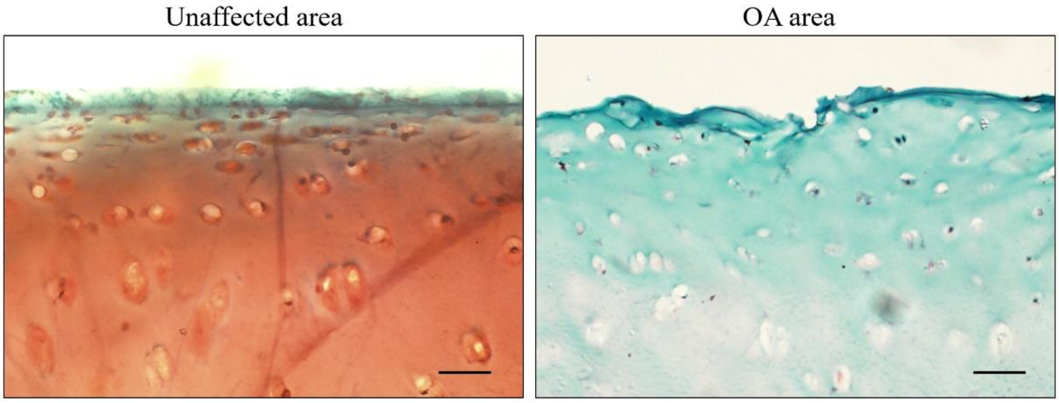


**Supplementary Fig. 9. Safranin O/Fast Green staining of unaffected and OA areas of human cartilage specimens.** Scale bar: 50 μm.

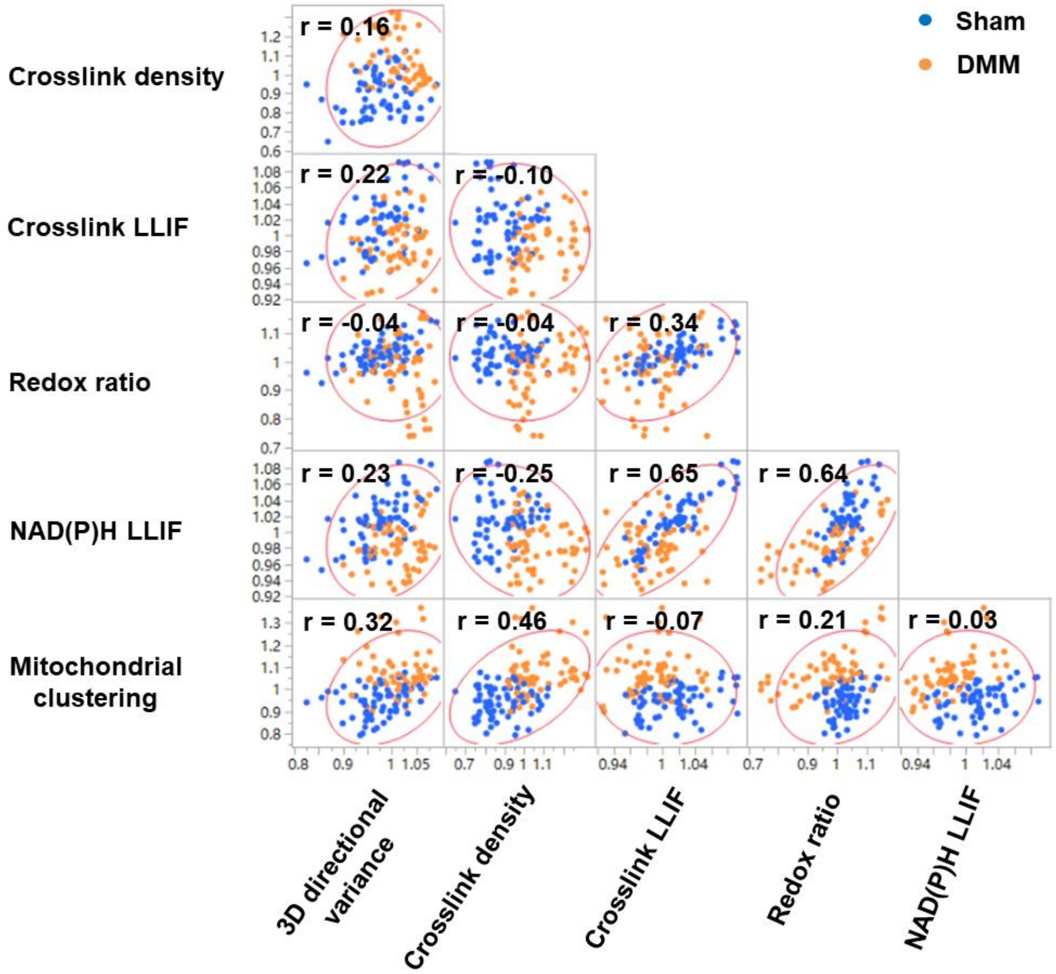


**Supplementary Fig. 10. Multicollinearity diagnostics through pairwise correlations from mouse week 10 data**. All the correlation coefficients (marked in each panel) satisfy the equation: $|r| < 0.7$ , indicating no offending variables and independence among these six variables.

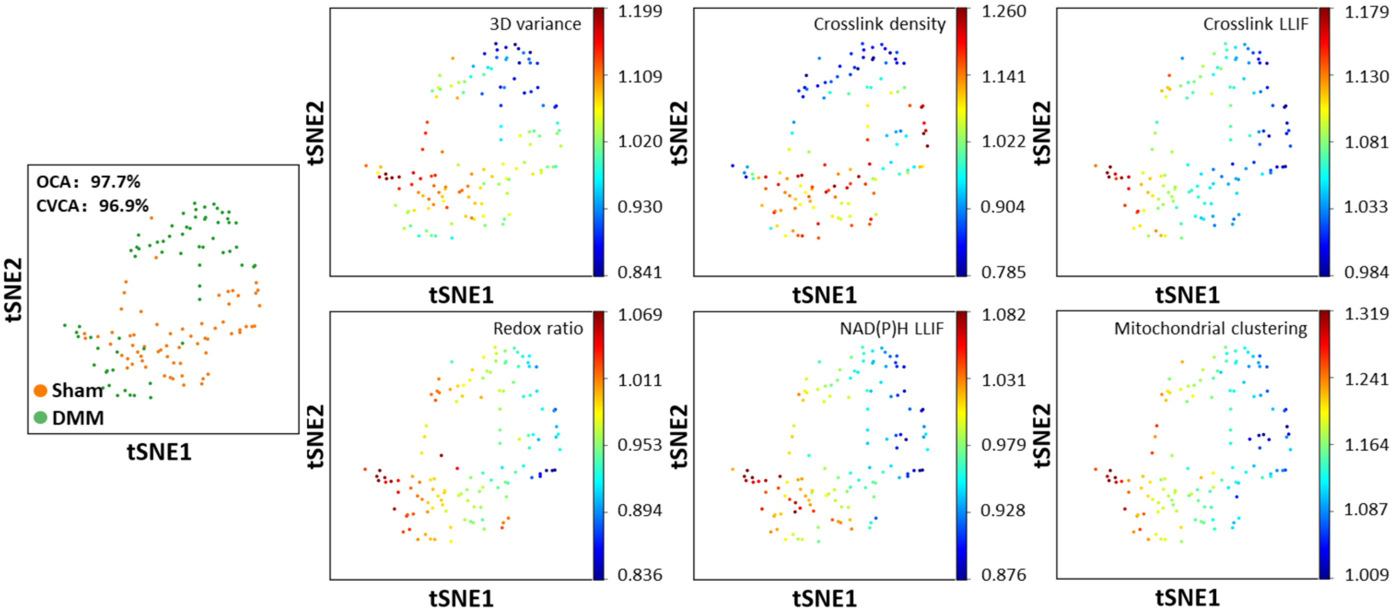


**Supplementary Fig. 11. Classification between DMM and sham in mouse model at week 1 post-surgery using viSNE analysis.** The viSNE map (left most) exhibits classification between the two groups, along with distribution maps of 3D directional variance, crosslink density, crosslink LLIF, redox ratio, NAD(P)H LLIF, and mitochondrial clustering which show the gradient of each metric across different groups. OCA and CVCA values are marked in the viSNE map.

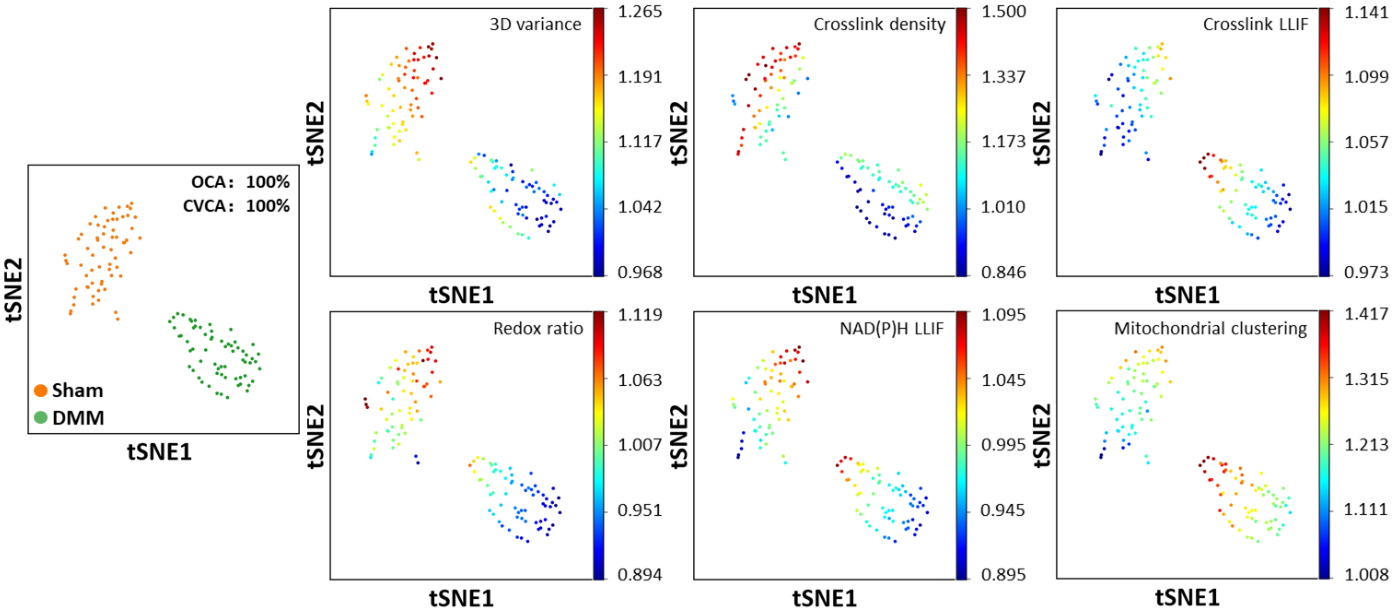


**Supplementary Fig. 12. Classification between DMM and sham in mouse model at week 2 post-surgery using viSNE analysis.** The viSNE map (left most) exhibits classification between the two groups, along with distribution maps of 3D directional variance, crosslink density, crosslink LLIF, redox ratio, NAD(P)H LLIF, and mitochondrial clustering which show the gradient of each metric across different groups. OCA and CVCA values are marked in the viSNE map.

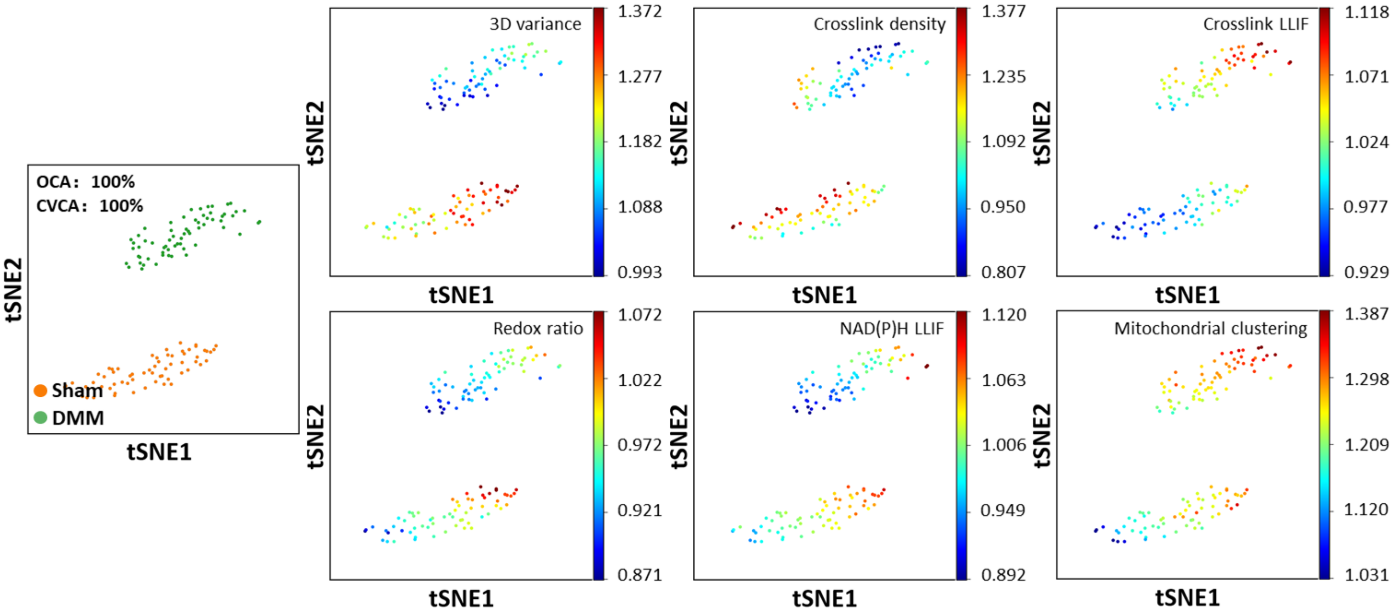


**Supplementary Fig. 13. Classification between DMM and sham in mouse model at week 3 post-surgery using viSNE analysis.** The viSNE map (left most) exhibits classification between the two groups, along with distribution maps of 3D directional variance, crosslink density, crosslink LLIF, redox ratio, NAD(P)H LLIF, and mitochondrial clustering which show the gradient of each metric across different groups. OCA and CVCA values are marked in the viSNE map.

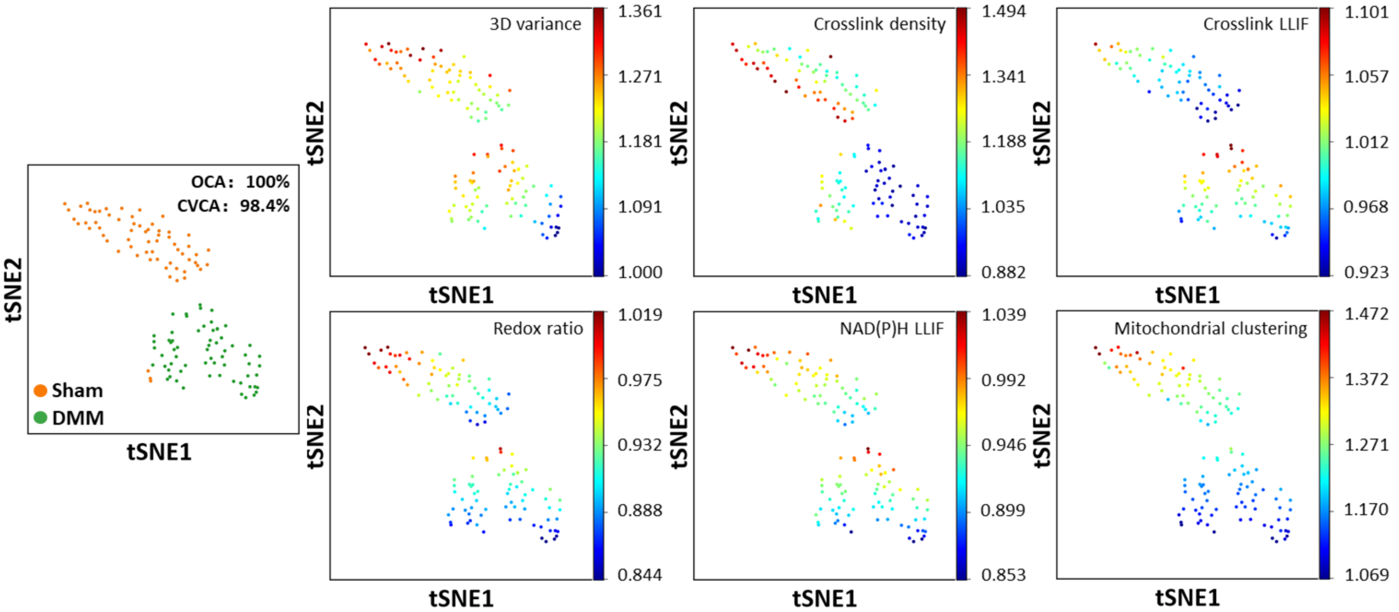


**Supplementary Fig. 14. Classification between DMM and sham in mouse model at week 7 post-surgery using viSNE analysis.** The viSNE map (left most) exhibits classification between the two groups, along with distribution maps of 3D directional variance, crosslink density, crosslink LLIF, redox ratio, NAD(P)H LLIF, and mitochondrial clustering which show the gradient of each metric across different groups. OCA and CVCA values are marked in the viSNE map.

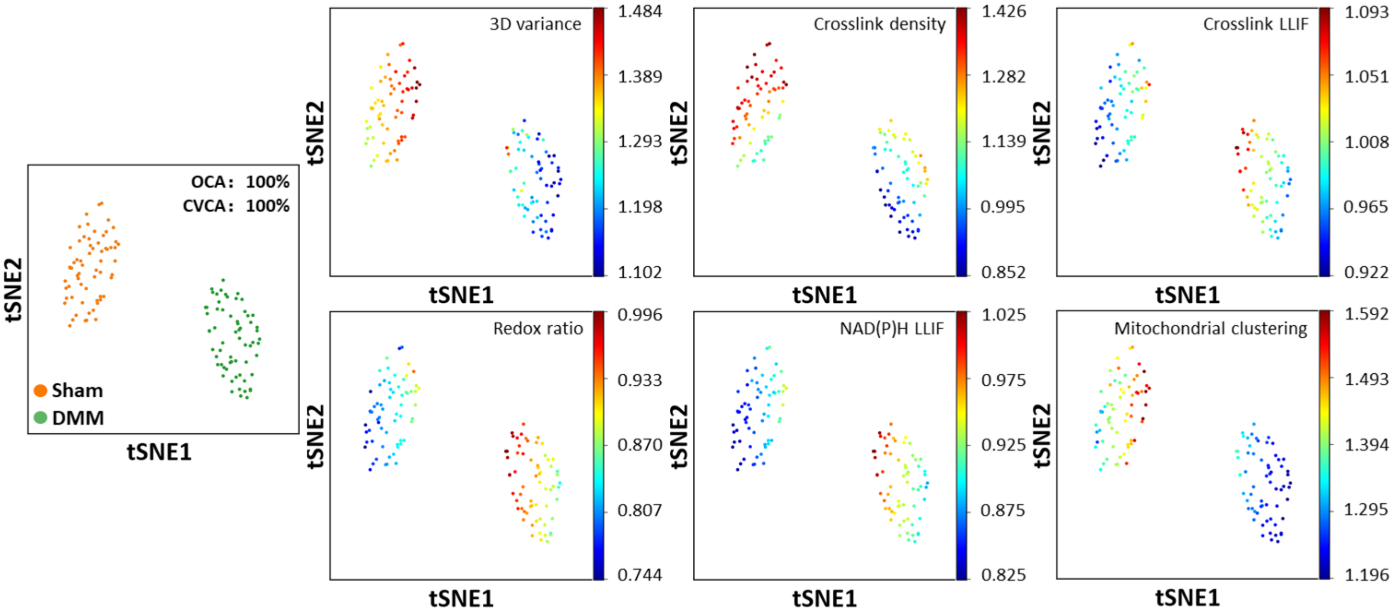


**Supplementary Fig. 15. Classification between DMM and sham in mouse model at week 10 post-surgery using viSNE analysis.** The viSNE map (left most) exhibits classification between the two groups, along with distribution maps of 3D directional variance, crosslink density, crosslink LLIF, redox ratio, NAD(P)H LLIF, and mitochondrial clustering which show the gradient of each metric across different groups. OCA and CVCA values are marked in the viSNE map.

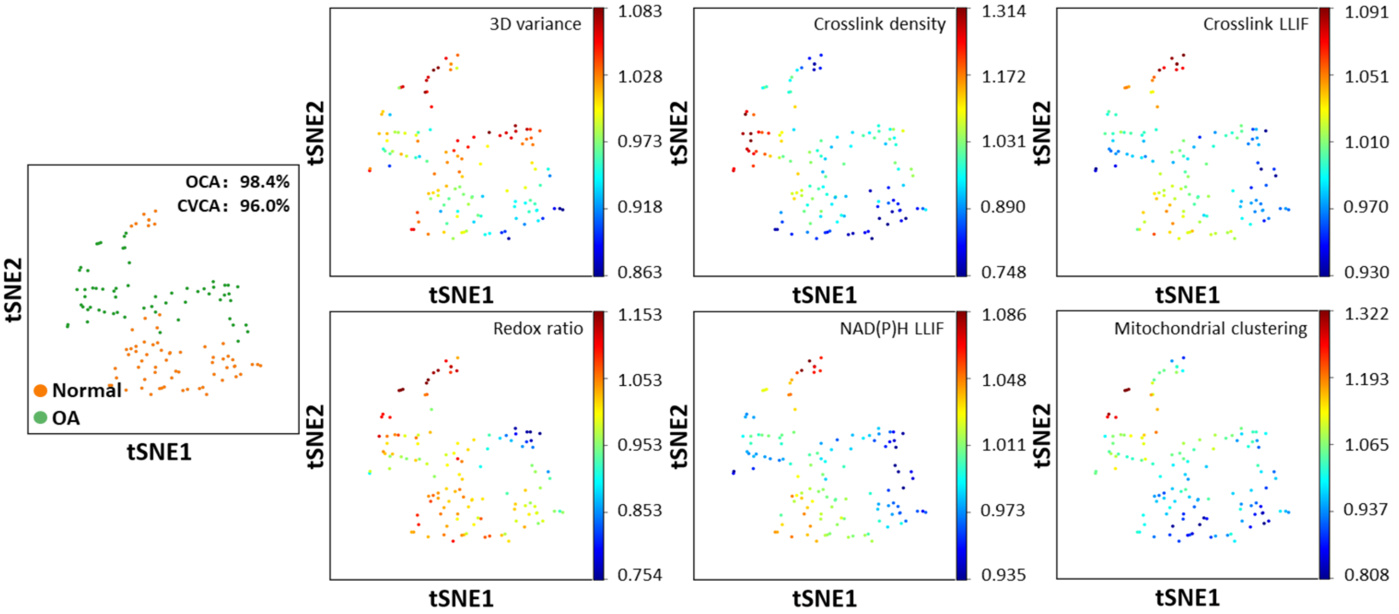


**Supplementary Fig. 16. Classification between normal and OA human specimens using viSNE analysis.** The viSNE map (left most) exhibits classification between the two groups, along with distribution maps of 3D directional variance, crosslink density, crosslink LLIF, redox ratio, NAD(P)H LLIF, and mitochondrial clustering which show the gradient of each metric across different groups. OCA and CVCA values are marked in the viSNE map.

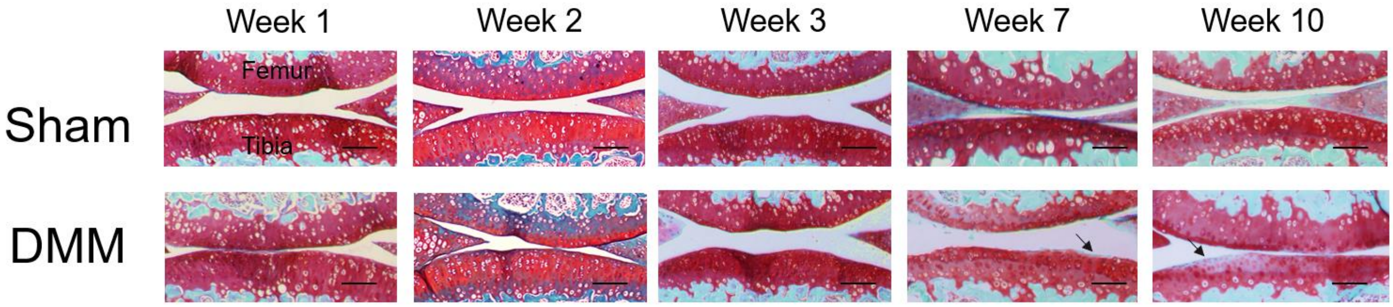


**Supplementary Fig. 17. Safranin O/Fast Green staining of mouse knee sections at 1, 2, 3, 7, and 10 weeks post DMM surgery.** Arrows point to locations with obvious matrix loss. Scale bar: 400 μm.

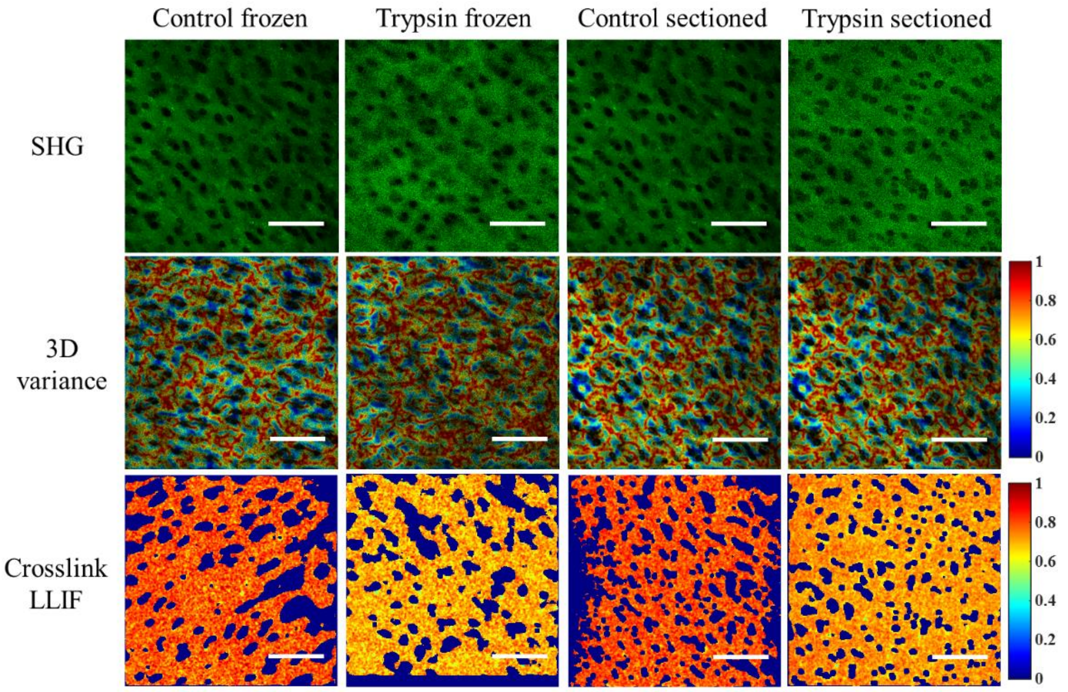


**Supplementary Fig. 18. Representative maps of SHG intensity, 3D directional variance, and crosslink LLIF obtained from frozen and cryosectioned control or trypsin-treated porcine cartilage tissues.** Scale bar: 50 μm.

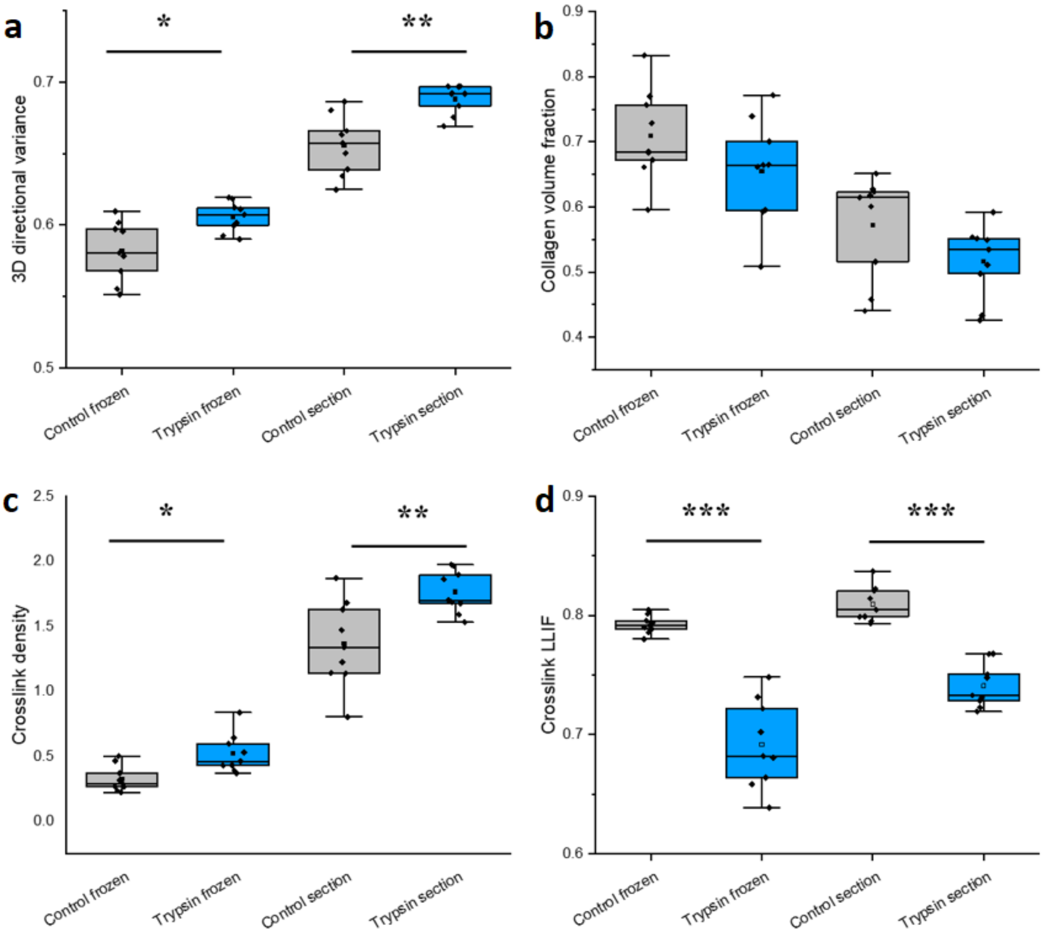


**Supplementary Fig. 19. Impact of sample processing on collagen-related optical metrics.** Field-based boxplots of **a**, 3D directional variance, **b**, collagen volume fraction, **c**, crosslink density, and **d**, crosslink LLIF. *, $p < 0.05$, **, $p < 0.01$ and ***, $p < 0.001$.

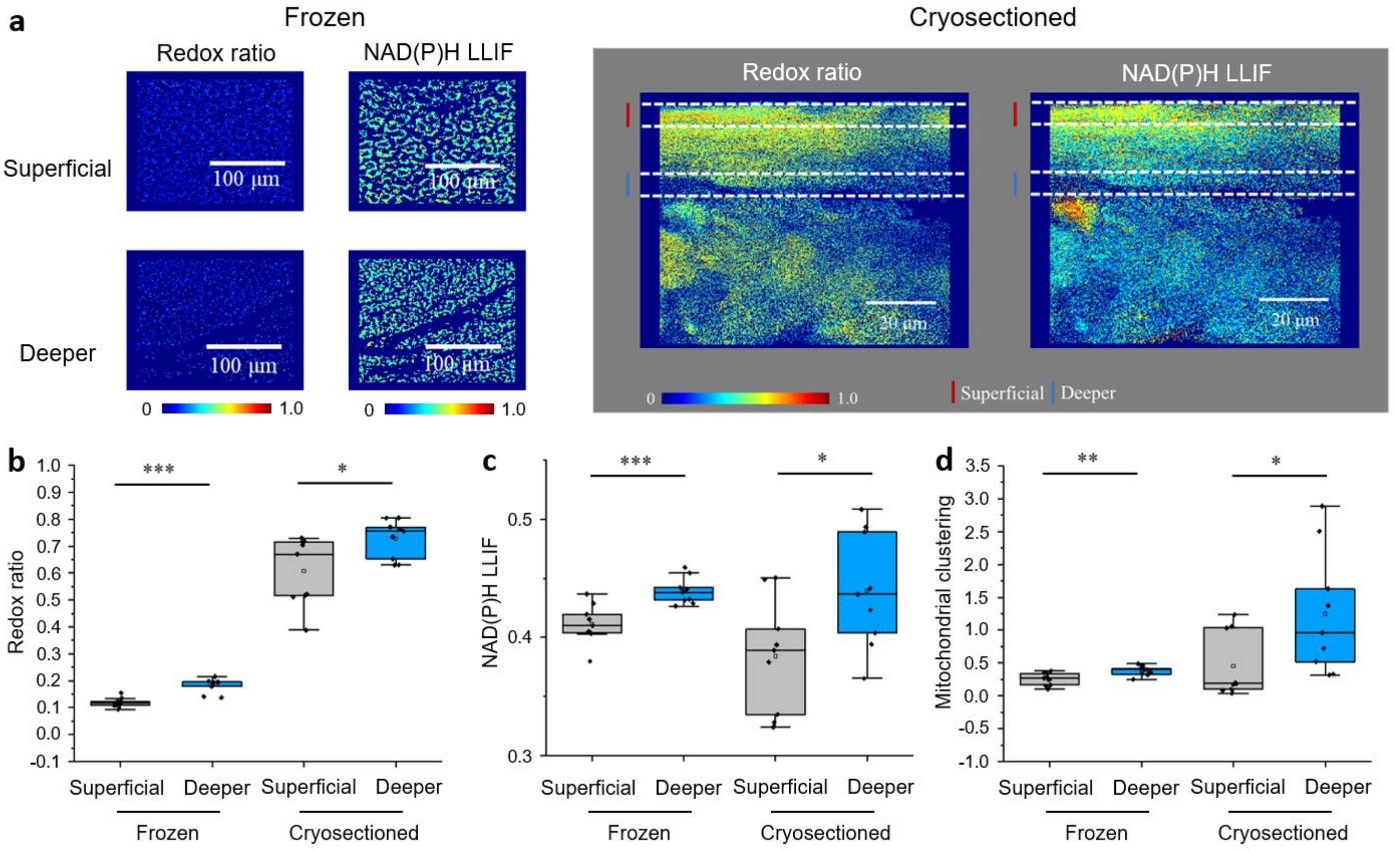


**Supplementary Fig. 20. Impact of sample processing on metabolic metrics in superficial and deeper regions of rodent epithelial tissues. a**, Representative maps of redox ratio and NAD(P)H LLIF obtained from frozen and cryosectioned rodent young epithelial tissues. Field-based boxplots of **b**, redox ratio, **c**, NAD(P)H LLIF, and **d**, mitochondrial clustering. *, $p < 0.05$, **, $p < 0.01$ and ***, $p < 0.001$.

**Supplementary Table:**

**Supplementary Table 1. Ranking of the optical parameter according to its ability of distinguishing different groups**

| No. | Week 1 | Week 2 | Week 3 | Week 7 | Week 10 | Human |
|---|---|---|---|---|---|---|
| 1 | 3D directional variance | 3D directional variance | 3D directional variance | Mitochondrial clustering | 3D directional variance | Mitochondrial clustering |
| 2 | Crosslink density | Redox ratio | Crosslink LLIF | Crosslink density | Mitochondrial clustering* | Crosslink density |
| 3 | Crosslink LLIF | Crosslink density | Crosslink density | Crosslink LLIF | Redox ratio* | NAD(P)H LLIF |
| 4 | Redox ratio* | NAD(P)H LLIF | NAD(P)H LLIF* | Redox ratio | NAD(P)H LLIF | Crosslink LLIF |
| 5 | Mitochondrial clustering* | Mitochondrial clustering | Mitochondrial clustering* | NAD(P)H LLIF* | Crosslink density | 3D directional variance* |
| 6 | NAD(P)H LLIF | Crosslink LLIF | Redox ratio | 3D directional variance* | Crosslink LLIF | Redox ratio* |

* These metrics have the same discriminative power
