# Supplementary material for: Label-free, multi-parametric assessments of cell metabolism and matrix remodeling within human and early-stage murine osteoarthritic articular cartilage
Source: Commun Biol. 2023 Apr 13;6:405. doi: 10.1038/s42003-023-04738-w (PMC10102009; doi:10.1038/s42003-023-04738-w)
Supplement: Supplementary file 2 — Description of Additional Supplementary Files [file 42003_2023_4738_MOESM2_ESM.pdf]

Description of Additional Supplementary Files

File Name: Supplementary Data 1

Description: The source data behind the graphs in the paper.

File Name: Supplementary video 1

Description: 3D images of different contrasts from the four different channels. These contrasts include: NAD(P)H image, FAD image, TPEF signal with 755 nm excitation and 525 nm emission, and SHG image of collagen fibers. Cartilage from the tibia dominates the image field, with cartilage from the femur, with a gap between them, also included. Scale bar: 50 µm

File Name: Supplementary video 2

Description: Dynamic changes of the metabolic activity induced by OA. The gray hue corresponds to OA data from mouse or human specimens, and the other hues correspond to the established database summarizing metabolic activity in response to different perturbations.
