# Supplementary material for: Label-free, multi-parametric assessments of cell metabolism and matrix remodeling within human and early-stage murine osteoarthritic articular cartilage
Source: Commun Biol. 2023 Apr 13;6:405. doi: 10.1038/s42003-023-04738-w (PMC10102009; doi:10.1038/s42003-023-04738-w)
Supplement: Supplementary file 6 — Reporting Summary [file 42003_2023_4738_MOESM6_ESM.pdf]

nature portfolio

Corresponding author(s): Irene Georgakoudi

Last updated by author(s): Feb 24, 2023

# Reporting Summary

Nature Portfolio wishes to improve the reproducibility of the work that we publish. This form provides structure for consistency and transparency in reporting. For further information on Nature Portfolio policies, see our Editorial Policies and the Editorial Policy Checklist.

## Statistics

For all statistical analyses, confirm that the following items are present in the figure legend, table legend, main text, or Methods section.

| n/a | Confirmed | |
|---|---|---|
| ☐ | ☒ | The exact sample size (*n*) for each experimental group/condition, given as a discrete number and unit of measurement |
| ☐ | ☒ | A statement on whether measurements were taken from distinct samples or whether the same sample was measured repeatedly |
| ☐ | ☒ | The statistical test(s) used AND whether they are one- or two-sided<br>*Only common tests should be described solely by name; describe more complex techniques in the Methods section.* |
| ☐ | ☒ | A description of all covariates tested |
| ☐ | ☒ | A description of any assumptions or corrections, such as tests of normality and adjustment for multiple comparisons |
| ☐ | ☒ | A full description of the statistical parameters including central tendency (e.g. means) or other basic estimates (e.g. regression coefficient) AND variation (e.g. standard deviation) or associated estimates of uncertainty (e.g. confidence intervals) |
| ☐ | ☒ | For null hypothesis testing, the test statistic (e.g. *F*, *t*, *r*) with confidence intervals, effect sizes, degrees of freedom and *P* value noted<br>*Give P values as exact values whenever suitable.* |
| ☒ | ☐ | For Bayesian analysis, information on the choice of priors and Markov chain Monte Carlo settings |
| ☒ | ☐ | For hierarchical and complex designs, identification of the appropriate level for tests and full reporting of outcomes |
| ☐ | ☒ | Estimates of effect sizes (e.g. Cohen's *d*, Pearson's *r*), indicating how they were calculated |

*Our web collection on statistics for biologists contains articles on many of the points above.*

## Software and code

Policy information about availability of computer code

| | |
|---|---|
| Data collection | Data were collected using a Leica TCS SP8 confocal microscope equipped with a tunable (680-1300 nm) fs laser (InSight Deep See; Spectra Physics; Mountain View, CA). Also, picrosirius red staining images of mouse cartilage samples were viewed using polarized light on the Zeiss Axioskop2 microscope. |
| Data analysis | Data were analyzed using custom code which was developed in MATLAB and available for download at: https://engineering.tufts.edu/bme/georgakoudi/publications, as well as the Zenodo platform. |

For manuscripts utilizing custom algorithms or software that are central to the research but not yet described in published literature, software must be made available to editors and reviewers. We strongly encourage code deposition in a community repository (e.g. GitHub). See the Nature Portfolio guidelines for submitting code & software for further information.

## Data

Policy information about availability of data

All manuscripts must include a data availability statement. This statement should provide the following information, where applicable:

- Accession codes, unique identifiers, or web links for publicly available datasets
- A description of any restrictions on data availability
- For clinical datasets or third party data, please ensure that the statement adheres to our policy

The data that support the findings of this study are available from the corresponding authors upon request.

# Field-specific reporting

Please select the one below that is the best fit for your research. If you are not sure, read the appropriate sections before making your selection.

☒ Life sciences ☐ Behavioural & social sciences ☐ Ecological, evolutionary & environmental sciences

For a reference copy of the document with all sections, see nature.com/documents/nr-reporting-summary-flat.pdf

# Life sciences study design

All studies must disclose on these points even when the disclosure is negative.

| | |
|---|---|
| Sample size | For mice DMM samples, we collected data from knee joints at 1, 2, 3, 7 and 10 weeks post-surgery, from four mice at each time point. For human samples, we collected data from human articular cartilage samples from the tibial plateaus of three patients undergone total knee replacement surgery for OA at Tufts Medical Center. For mouse MIA samples, we collected data from three mice for each group (i.e., MIA or control). For both porcine cartilage and rodent epithelial tissues, n = 3 animals. |
| Data exclusions | No data were excluded from the analysis. |
| Replication | The experiments in this study were performed using at least 3 independent replicates to ensure robustness. Attempts at replication in this study were successful. |
| Randomization | Tissue slides from multiple replicates were randomly selected and imaged for analysis. |
| Blinding | Blinding was not applicable to this study, because this study tries to compare the cellular metabolism and collagen organization characteristics between normal and osteoarthritis articular cartilage. |

# Reporting for specific materials, systems and methods

We require information from authors about some types of materials, experimental systems and methods used in many studies. Here, indicate whether each material, system or method listed is relevant to your study. If you are not sure if a list item applies to your research, read the appropriate section before selecting a response.

## Materials & experimental systems

| n/a | Involved in the study | |
|---|---|---|
| ☒ | ☐ | Antibodies |
| ☒ | ☐ | Eukaryotic cell lines |
| ☒ | ☐ | Palaeontology and archaeology |
| ☐ | ☒ | Animals and other organisms |
| ☐ | ☒ | Human research participants |
| ☒ | ☐ | Clinical data |
| ☒ | ☐ | Dual use research of concern |

## Methods

| n/a | Involved in the study | |
|---|---|---|
| ☒ | ☐ | ChIP-seq |
| ☒ | ☐ | Flow cytometry |
| ☒ | ☐ | MRI-based neuroimaging |

## Animals and other organisms

Policy information about studies involving animals; ARRIVE guidelines recommended for reporting animal research

| | |
|---|---|
| Laboratory animals | Laboratory animals: 8-week-old male BALB/c mice (Taconic, NY, USA); 18-week-old male C57B/L6 mice. |
| Wild animals | The study did not involve wild animals. |
| Field-collected samples | To establish the DMM model, 8-week-old male BALB/c mice (Taconic, NY, USA) were housed in individual cages and acclimated at room temperature (22–25 °C). Mice were given free access to standard laboratory chow and tap water. Regarding the osteoarthritis-inducing surgery, the right knee joint was opened along the medial border of the patellar ligament and the medial meniscotibial ligament was severed. The left knee joint received a sham surgery, in which the ligament was exposed, while not severed. The knee joints from mice undergoing no surgery served as non-surgery controls. After experiment, the mice were euthanized by isoflurane anesthesia followed by cervical dislocation.<br>To establish the MIA model, MIA (5 µg in 5 µL PBS) was directly injected into the knee joint of male C57B/L6 mice (18 weeks old). PBS injection served as a control. Injections took place daily for 7 days, when the knee joint was harvested, paraffin embedded and sectioned for collagen organization analysis. |
| Ethics oversight | Tufts University Institutional Animal Care and Use Committee (IACUC) |

Note that full information on the approval of the study protocol must also be provided in the manuscript.

## Human research participants

Policy information about studies involving human research participants

| | |
|---|---|
| Population characteristics | We collected human articular cartilage samples from the tibial plateaus of three patients undergone total knee replacement surgery for osteoarthritis at Tufts Medical Center. The age, sex and the Mankin scores of these donors were 63 (female, score: 7), 65 (female, score: 9) and 85 (male, score: 8), respectively. To validate the affected areas of human cartilage specimens, we also collected samples from human cartilage donors from National Disease Research Interchange (NDRI) and performed Safranin O/Fast Green staining. |
| Recruitment | Inclusion criteria were patients who underwent total knee replacement surgery for osteoarthritis. No potential self-selection bias or any other biases that may impact results. |
| Ethics oversight | Tufts Institutional Review Board (IRB) |

Note that full information on the approval of the study protocol must also be provided in the manuscript.
